# Supplementary material for: Assessment of the compound impact of sea level rise, land subsidence and storm surge under climate change in ShangHai
Source: PLoS One. 2026 Mar 18;21(3):e0343974. doi: 10.1371/journal.pone.0343974 (PMC12998868; doi:10.1371/journal.pone.0343974)
Supplement: Attachment — Submitted filename: PONE-D-25-32386_ln_ R1.pdf [file pone.0343974.s001.pdf]

Assessment of the compound impact of sea level rise, land subsidence and storm surge under climate change

Bing Liang[1, 2], Guoqing Shi[3], Yuexi Wu[4], Yuxuan Zhu[4], Mark Wang[2], Zhonggen Sun[3]

[1] Business School,Wuxi Taihu University,Wuxi 214063,China

[2] Centre for contemporary Chinese studies， University of Melbourne,Parkville,VIC3010, Australia

[3] National Research Center for Resettlement, Hohai University, Nanjing 211100, China

[4] College of Geography and Remote Sensing, Hohai University,Nanjing 210098, China

*** Correspondence:** Bing Liang, bliang1@126.com; Guoqing Shi, gshi@hhu.edu.cn.

Abstract: Global climate change-induced sea level rise has emerged as a critical environmental challenge for coastal cities in the 21st century. Shanghai, China's economic, financial, and shipping hub, faces significantly amplified inundation risks in its coastal areas due to the compounding effects of sea level rise, land subsidence, and storm surges. This study constructs a multi-scenario simulation framework using IPCC AR6 sea level projection data, land subsidence monitoring records, and historical storm surge data to evaluate the impacts of three scenarios on future inundation risks: sea level rise alone (Scenario 1), sea level rise combined with land subsidence (Scenario 2), and sea level rise coupled with land subsidence and storm surges (Scenario 3). Leveraging ARIMA time-series modeling, GIS spatial analysis, and numerical simulations, the study predicts relative sea level rise and inundation extents for 2050, 2070, and 2100. Results indicate that Shanghai's relative sea level rise rate far exceeds the global average, with land subsidence and storm surges synergistically amplifying disaster risks in low-lying coastal zones. Under Scenario 1, the projected inundation area reaches 361.32 km² by 2100. Scenario 2 increases this area to 460.97 km², while Scenario 3 (including storm surges) shows a dramatic escalation to 1,331.91 km² by 2100—a surge of 870.94 km² compared to Scenario 2—highlighting the dominant role of storm surges in extreme weather events. Spatial analysis identifies Chongming District, Pudong New Area, and Fengxian District as high-risk zones, with Chongming Island being the most severely affected (54.5% inundation by 2100). This study elucidates the compound impact mechanisms of sea level rise, land subsidence, and storm surges in Shanghai, providing a scientific foundation for coastal disaster mitigation and adaptive urban management. Recommendations include enhancing coastal flood defenses, optimizing land-use planning, improving extreme weather early-warning systems, and fostering international collaboration and technological innovation to bolster urban resilience against climate risks.

Keywords: climate change, sea level rise, land subsidence, storm surge, compound effects,

inundation risk, Shanghai

1. Introduction

Global climate change-induced sea level rise (SLR) has become one of the most critical environmental challenges of the 21st century, posing severe threats to the sustainable development of coastal cities worldwide[1,2]. The Intergovernmental Panel on Climate Change (IPCC) Sixth Assessment Report (AR6) indicates that under global warming, global sea levels will continue to rise in the coming decades, potentially reaching 0.5–1.0 meters above pre-industrial levels by the end of the 21st centur[3]. Against this backdrop, coastal regions face significant challenges to ecological security, socioeconomic stability, and infrastructure resilience[4]. However, coastal cities are not only threatened by SLR but also by the compound effects of land subsidence and storm surges[5,6]. These three factors interact synergistically, creating a "rising seas and sinking land" phenomenon that significantly amplifies disaster risks in coastal urban areas.

Land subsidence represents another critical challenge for coastal cities, particularly in regions with complex geological conditions, excessive groundwater extraction, and rapid urbanization[7]. Cities such as Shanghai, Tokyo, and Jakarta have experienced pronounced subsidence in recent years, accelerating relative sea level rise rates far beyond the global average and exacerbating risks of flooding and salinization in coastal zones[8]. Furthermore, storm surges—intensified by global warming and SLR—are increasing in both frequency and severity, further jeopardizing coastal safety[9].

Despite extensive research on SLR, land subsidence, and storm surges individually, current studies predominantly focus on single-factor analyses, with limited systematic evaluation of their compound impacts. This study aims to address this gap by integrating multi-source remote sensing data, climate model projections, and numerical simulations to comprehensively assess the combined effects of SLR, land subsidence, and storm surges. It further investigates their synergistic mechanisms in driving coastal disaster risks. The findings are intended to provide scientific foundations for disaster mitigation and adaptive policymaking in coastal cities, offering critical decision-support for global climate resilience efforts.

2. Literature Review

The primary drivers of sea level rise (SLR) include thermal expansion, ice sheet melting, and glacier retreat[10,11,12]. Recent studies highlight significant regional disparities in SLR impacts on coastal zones[13]. For instance, Rahmstorf [14]emphasized that 21st-century SLR will disproportionately threaten cities in East Asia, Southeast Asia, and the eastern coast of

North America. In China, regions such as the Yangtze River Delta, Pearl River Delta, and Bohai Bay exhibit relative sea level rise rates exceeding the global average, partly due to regional climatic and hydrological dynamics[15].

Land subsidence, widely documented in coastal cities globally, has emerged as a critical amplifier of SLR impacts[16]. Jakarta, for example, experiences subsidence rates as high as 25 cm/yr—far surpassing global SLR rates[17]. Chinese coastal cities like Shanghai and Tianjin also face persistent subsidence driven by groundwater extraction and soft soil compaction. Research indicates that subsidence exacerbates coastal flooding risks and compromises the efficacy of flood defense systems[18], underscoring the need to integrate subsidence into relative sea level assessments for coastal risk management.

Storm surge frequency and intensity have increased globally, particularly in the North Atlantic and western Pacific. Studies demonstrate that SLR amplifies storm surge impacts, rendering previously secure coastal areas increasingly vulnerable to extreme events[19]. For example, Kirezci et al.[20]projected that under the RCP8.5 high-emissions scenario, storm surge-induced flooding could expand by nearly 50% globally by 2100. Furthermore, the interplay between storm surges and subsidence magnifies disaster risks, as evidenced by Hurricane Sandy's unprecedented flooding in New York (2012), where cumulative relative sea level rise and subsidence exacerbated impacts .

While existing research elucidates the individual effects of SLR, subsidence, and storm surges, systematic evaluations of their compound impacts remain limited. Moreover, most studies rely on historical regression analyses, with few projecting future scenarios. Addressing these gaps, this study contributes by: Developing a multi-scenario assessment framework: Integrating multi-source remote sensing data, climate models, and numerical simulations to quantify the compound effects of SLR, subsidence, and storm surges on coastal disaster risks.Focusing on Shanghai as a case study, leveraging long-term observational data to analyze spatiotemporal patterns of these factors and assess their implications for urban resilience.

This research not only advances understanding of compound climate-hazard interactions but also provides actionable insights for adaptive coastal governance, offering policy-relevant guidance for global sustainable development efforts.

3 Methods

3.1 Study Area

Shanghai, located in eastern China (120°51′E–122°12′E, 30°40′N–31°53′N), serves as a vital coastal gateway city. Situated on the western Pacific coast near the Yangtze River estuary, it lies at the confluence of the Yangtze River and the East China Sea. Bordered by the East China

Sea to the east, Hangzhou Bay to the south, and Jiangsu and Zhejiang provinces to the west, Shanghai anchors China's most economically developed Yangtze River Delta region. Its strategic location has established the city as a global economic, financial, trade, and shipping hub.

Shanghai's terrain is predominantly flat, forming part of the Yangtze River Delta alluvial plain, with an average elevation of approximately 2.19 meters. The land slopes gently from east to west. While low-lying plains dominate, the southwestern area features minor hills and mountains, including Dajinshan—the highest natural point at 103.4 meters above sea level. Although the flat terrain supports agriculture and urban expansion, it also exacerbates geological hazards such as land subsidence and flooding, particularly under escalating sea level rise and climate extremes.

Shanghai experiences a subtropical monsoon climate, characterized by distinct seasons, mild temperatures, and abundant humidity. The annual average temperature is around 15.8°C, with precipitation exceeding 1,200 millimeters, concentrated during the plum rain season. Typhoons and heavy rainfall are frequent in spring and summer, posing challenges to urban infrastructure. The city's coastal location subjects it to complex hydrological conditions influenced by the East China Sea and Yangtze River. The dense river network, including the Huangpu and Wusong Rivers, not only provides abundant water resources and navigation convenience but also shapes a unique waterscape.

Administratively, Shanghai comprises urban districts and three offshore islands: Chongming, Changxing, and Hengsha. Chongming Island, China's third-largest island, holds critical ecological significance. As a coastal metropolis, Shanghai has abundant coastline resources. Its coastal wetlands and estuary areas play a crucial role in environmental protection. Chongming Island's wetlands, designated as one of the globally important wetland reserves, are especially significant for bird migration and ecological balance.

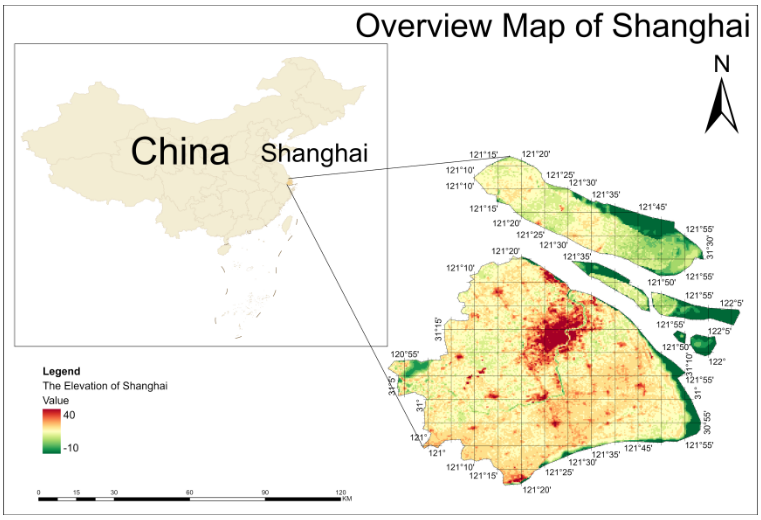

Fig.1 Overview map of Shanghai

3.2 Data Sources

① Sea level rise (SLR) projections: Derived from the IPCC AR6 dataset accessed via NASA's Sea Level Change Portal (SeaLevelProjectionTool–NASASeaLevelChangePortal).

② Land subsidence data: Obtained from the Shanghai Geological Environment Bulletin (2009–2023).

③ Digital Elevation Model (DEM) for Shanghai: Sourced from the GEBCO website (https://www.gebco.net/data_and_products/gridded_bathymetry_data/).

3.3 Methodology

3.3.1 Scenario Design

Sea-level rise is divided into absolute and relative types, which describe changes based on different benchmarks. Absolute sea-level rise refers to the increase in global or regional sea levels relative to a fixed reference, usually the sea level itself. In contrast, relative sea-level rise indicates the rise in sea level at a specific location relative to the local land surface. They have different reference benchmarks. Absolute sea-level rise is affected only by global factors, while relative sea-level rise is influenced by both global and local factors[21]. Therefore, this study incorporates local factors like land subsidence and storm surges when examining sea-level rise in Shanghai.

To account for localized impacts, this study evaluates three scenarios for Shanghai:

Scenario 1: Absolute SLR only.

Scenario 2: Absolute SLR combined with land subsidence.

Scenario 3: Absolute SLR combined with both land subsidence and storm surges.

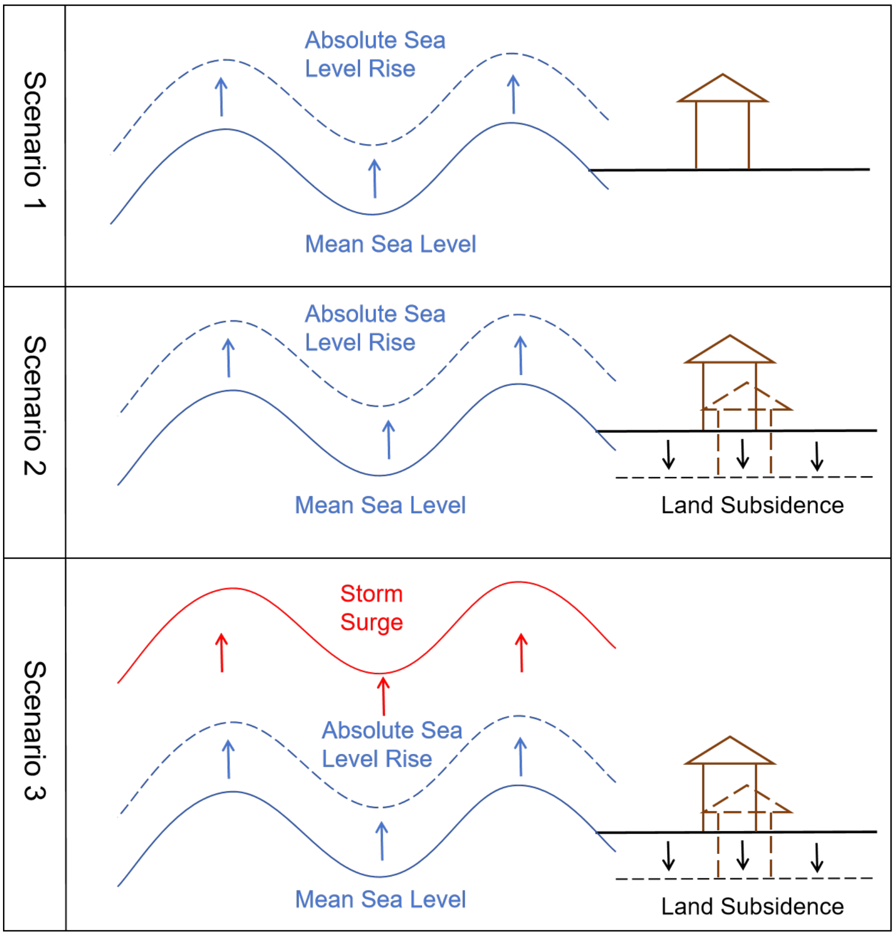

Fig.2 Schematic diagram of different scenario modes

3.3.2 SLR Projection Methodology

The sea level rise (SLR) projections for Shanghai (2050, 2070, 2100) were derived using prediction data from the IPCC AR6 report and the NASA Sea Level Change Portal, along with predictive calculation formulas. On the portal, the prediction data from the nearest coastal station to Shanghai was used as the basis for Shanghai's sea level rise projections. Under the SSP2-4.5 scenario, SLR projections for Shanghai were obtained for the years 2050, 2070, and 2100.

3.3.3 Land Subsidence Prediction Methodology

Time series analysis is a statistical, mathematical, and econometric technique for processing sequential data to identify patterns, trends, and periodicities, enabling forecasting and inference[22]. Among various methods, the ARIMA model (Autoregressive Integrated Moving Average model) is a widely adopted classical approach for time series analysis. This model integrates three core components: autoregression (AR), differencing (I), and moving average (MA), making it suitable for analyzing diverse types of time series data.

The ARIMA model converts non-stationary time series into stationary ones through

differencing, then models and forecasts the data using AR and MA components. The general form of an ARIMA model is denoted as ARIMA(p, d, q), where: p: Number of autoregressive terms. d: Order of differencing to achieve stationarity. q: Number of moving average terms. The autoregressive (AR) component captures the linear relationship between a variable’s current value and its past values. The differencing (I) component addresses non-stationarity by removing trends and seasonality. The moving average (MA) component refines predictions using a weighted average of past forecast errors.

Specifically, the autoregressive (AR) part of the ARIMA model depicts the linear relation between a variable's current value and its past values, leveraging the dependency structure of historical data to forecast future data. The integrated (I) part addresses non - stationarity in time series. By applying appropriate differencing to the series, it attains stationarity, which is crucial for many real - world time series datasets. Finally, the moving average (MA) component enhances forecasting by using the weighted average of current and past prediction errors, thereby reducing uncertainty caused by random fluctuations. The formula is in the form of:

$$y_t = \mu + \sum_{i=1}^{p} \gamma_i y_{t-i} + \epsilon_t \quad (1)$$

Where yt is the current value, μ is a constant, γi are autoregressive coefficients, and ϵt is the error term.

Differencing (I): A critical step to stabilize non-stationary data involving d times of differencing.

Moving - Average (MA) model: Focuses on the accumulation of errors in autoregressive models. It forecasts current values via a linear combo of past white - noise errors. The formula is:

$$y_t = \mu + \sum_{i=1}^{q} \theta_i \epsilon_{t-i} + \epsilon_t \quad (2)$$

where θ_i are moving average coefficients.

In this study, the ARIMA model predicts land - subsidence in Shanghai. As data is available from 2009 - 2023, the land subsidence data is extrapolated backwards to 1995 to align with the absolute sea - level rise baseline. Then, data from 1995 - 2023 is used to forecast subsidence from 2024 - 2100. Finally, using 1995 - 2014 as the baseline, the cumulative subsidence is projected to.

3.3.4 Storm Surge Inundation Delineation

The inundation width (D) resulting from storm surges height (H) and nearshore average

slope (θ):

$$D = H/\tan\theta \qquad (3)$$

Where H is the storm surge height, θ is the nearshore average slope, D is the inundation width.

In this study, the slope of Shanghai was calculated using the city's DEM data and the ArcGIS Pro platform. The average slope within 2 km of the coastline was derived and used as the near - shore average slope. After determining the value of H, the value of D was calculated. All these processes were implemented in ArcGIS Pro for visualization.

3.3.5 Inundation Area Calculation Methodology

Using the ArcGIS Pro platform, the collected DEM data for Shanghai were reclassified according to the projected sea-level rise (SLR) values. The reclassified raster data were then converted into vector polygons through a "raster-to-polygon" operation. An area field was added to the attribute table, and geometric calculations were performed to derive the inundation area under each SLR scenario.

4. Results

4.1 Absolute Sea Level Rise Projections

Based on IPCC AR6 projections, the predicted absolute sea level rise (SLR) for Shanghai is summarized in Table 1.

Table 1 Forecast of absolute sea level rise in Shanghai (unit: m)

Year	Absolute sea level rise
2050	0.32
2070	0.49
2100	0.81

4.2 Land Subsidence Predictions

The predicted values of relative sea-level rise after incorporating land subsidence data are shown in Table 2, with the baseline period (1995–2014) aligned with absolute SLR reference years.

Table 2 Prediction of land subsidence in Shanghai (unit: m)

Year	Accumulation of land subsidence
2050年	0.28

2070年	0.35
2100年	0.43

4.3 Relative Sea Level Rise Projections

By integrating land subsidence data, the projected relative SLR values for Shanghai are detailed in Table 3.

Table 3 Forecast value of relative sea level rise in Shanghai (unit: m)

Year	Absolute sea level rise	Accumulation of land subsidence	relative sea level rise
2050	0.32	0.28	0.6
2070	0.49	0.35	0.84
2100	0.81	0.43	1.24

4.4 Storm Surge Inundation Projections

Using historical extremes from the Huangpu Park Station (peak storm surge height: 5.44 m), this study adopted a conservative surge height (H) of 6 m for scenario analysis. The nearshore average slope ($\alpha\alpha$) was calculated as 0.12° (tangent ≈ 0.0021), yielding a storm surge inundation width (D) of approximately 2,857.14 m via the formula $D=H/\alpha D=H/\alpha$. ArcGIS Pro simulations for Scenario 3 produced the inundation area projections listed in Table 4.

Table 4 Predicted inundation area of Shanghai in scenario 3 (unit: km^2)

Area	2050	2070	2100
Xuhui District	0.00	0.00	0.00
Jiading District	0.00	0.00	0.00
Songjiang District	0.00	0.00	0.00
Qingpu District	0.00	0.00	0.00
Putuo District	0.00	0.00	0.00
Minhang District	0.00	0.00	0.00
Jinshan District	47.15	47.15	55.42
Chongming District	697.02	697.02	725.28
Baoshan District	48.07	48.07	55.69
Pudong New Area	387.95	387.95	393.17
Yangpu District	0.00	0.00	0.00
Hongkou District	0.00	0.00	0.00
Jing'an District	0.00	0.00	0.00

Area	2050	2070	2100
Huangpu District	0.00	0.00	0.00
Fengxian District	100.88	100.88	102.35
Changning District	0.00	0.00	0.00
Total	1281.07	1281.07	1331.91

4.5 Inundation Area Projections Across Scenarios

Three scenarios were simulated to assess future inundation trends in Shanghai for 2050, 2070, and 2100. GIS spatial analysis and numerical modeling revealed distinct inundation patterns (Figure 3).

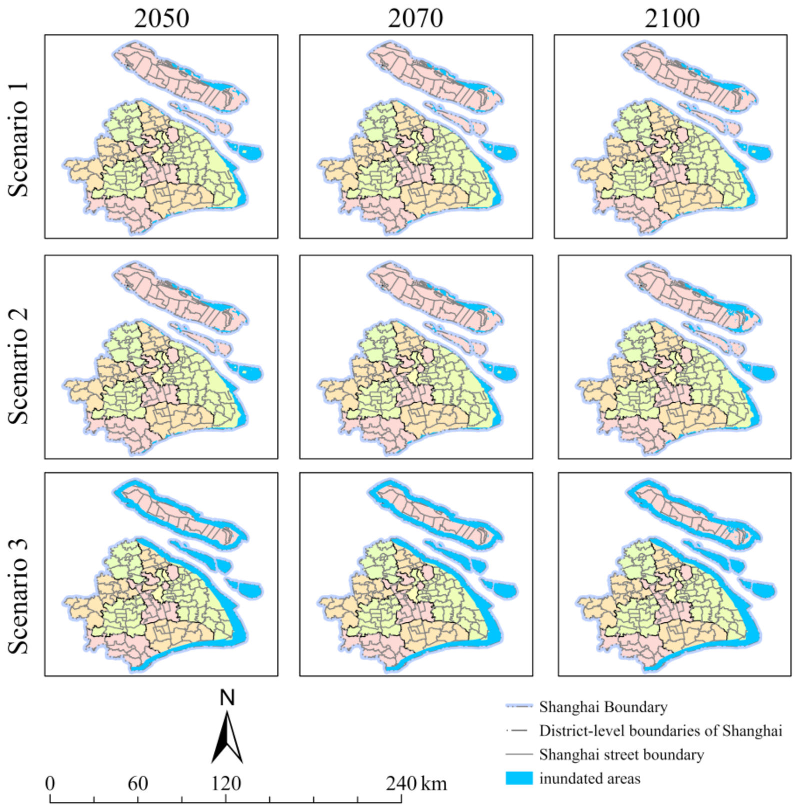

Fig.3 Inundation Forecast of Shanghai (2050, 2070, 2100)

Notably, inundation primarily expanded across eastern coastal zones, including Pudong New Area, Chongming District, and Fengxian District. Low-lying regions such as Chongming Dongtan (East Chongming Wetlands) and Nanhui Coastal Area were disproportionately impacted.

Table 5 Predicted value of inundation area under three scenarios (unit: km^2)

Scenario	2050	2060	2070	2080	2090	2100
Scenario 1	358.72	358.72	358.72	358.72	361.32	361.32
Scenario 2	358.72	358.72	358.72	358.72	460.97	460.97
Scenario 3	1281.07	1281.07	1281.07	1281.07	1331.90	1331.91

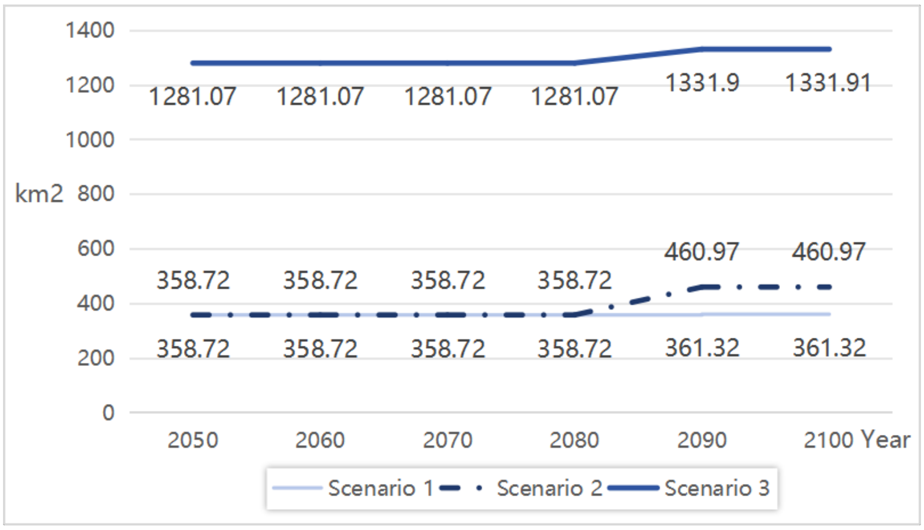

Fig.4 Prediction of inundation area under three scenarios (2050-2100)

From Table 5 and Figure 4, inundation areas under all three scenarios exhibit an increasing trend over time, with Scenario 3 (SLR + subsidence + storm surges) far exceeding the impacts of Scenarios 1 and 2. In 2050, inundation area under Scenario 1 (SLR alone) is 358.72 km², rising to 361.32 km² by 2100—a marginal increase. With subsidence added (Scenario 2), the inundation area reaches 460.97 km² by 2100, marking a 27.6% increase compared to Scenario 1, highlighting the cumulative amplifying role of subsidence. Most strikingly, Scenario 3 (including storm surges) causes inundation areas to surge from 1,281.07 km² in 2050 to 1,331.91 km² by 2100—a staggering increase of 870.94 km² over Scenario 2, underscoring the critical threat of storm surges under future extremes.

(1) Scenario 1 (SLR Only)

From 2050 to 2100, inundation areas remain relatively stable (~360 km²), concentrated in eastern Chongming District, coastal Fengxian District, and low-lying zones of Pudong New Area. While short-term impacts (2050–2070) are limited, gradual erosion in critical regions necessitates long-term monitoring (by 2100).

(2) Scenario 2 (SLR + Subsidence)

Post-2070, subsidence effects intensify, adding ~102.25 km² of inundation by 2100, primarily expanding into Pudong New Area, Baoshan District, and Fengxian District. Spatial variability in subsidence rates (e.g., 5–10 mm/yr in parts of Pudong) accelerates localized flooding. Cumulative subsidence exacerbates relative SLR, increasing long-term flood retention risks even without extreme weather.

(3) Scenario 3 (SLR + Subsidence + Storm Surges)

Storm surges dominate, driving inundation to 1,281.07 km² by 2050 and 1,331.91 km² by 2100—870.94 km² higher than Scenario 2. Figure 4 reveals extensive coastal inundation, particularly in Chongming Island, Jinshan coastal zones, and industrial areas of Pudong. Inland expansion threatens riverside urban districts (e.g., Yangpu, Hongkou) under extreme conditions, exposing critical infrastructure (ports, industrial parks) to severe risks. These results indicate that Shanghai's existing seawalls may prove inadequate in withstanding extreme storm surge events in the future

5. Discussion

5.1 Compound Effects of SLR and Subsidence Amplify Inundation Risks

Studies show that Shanghai, a coastal low - lying city, faces increased coastal flooding risks due to sea-level rise caused by global warming and land subsidence induced by long-term human activities, such as groundwater extraction[23].The combined effect of these two factors is not simply linear but exhibits a nonlinear amplification effect, particularly during extreme weather events, the extent and depth of flooding exceed expectations.

5.1.1 Mechanisms of Compound Impacts

Sea-level rise is mainly driven by thermal expansion and ice - sheet melting due to global warming[24], while land subsidence is more influenced by regional factors such as excessive groundwater extraction, geological activity, and urbanization.When both occur simultaneously, the rate of relative sea-level rise far surpasses the global average[25]. For instance, in this study's prediction under the IPCC SSP2-4.5 scenario, Shanghai's absolute sea-level rise by 2100 will reach 0.81 m, with about 0.43 m of cumulative land subsidence in the same period. After combining the two, the actual relative sea-level rise will be as high as 1.24 m. This indicates that even in the absence of additional extreme weather events, the long-term flooding risk in low-lying areas of Shanghai will significantly increase.

Moreover, land subsidence not only directly lowers the land surface but also undermines the stability of foundation structures, weakening the stability of urban flood - prevention and

drainage systems[26]. For example, ground subsidence can deform underground pipelines, reducing drainage capacity and worsening urban flooding during heavy rains. During storm surges, the lower land height allows tides to infiltrate further inland, expanding the flooded area and prolonging the duration of floodwater retention[27].

5.1.2 Spatial Heterogeneity

In Shanghai, varying ground - subsidence rates across districts lead to different relative sea - level rise impacts[28]. Pudong, Baoshan, and Chongming Districts, near the coast with soft soil, have high subsidence rates, worsening flood risks. By 2100, Chongming District's flooded area may reach 725.28 km², 54.5% of the city's total. Inland districts like Jing'an and Huangpu, elevated with slow subsidence, are less affected. Land use also affects flood risk[29]. Some industrial and residential zones, low - lying due to land - reclamation or foundation sinking, are more vulnerable to sea - level rise. For example, Pudong's coastal industrial parks face both subsidence and tidal threats, while Chongming Island, though with a lower subsidence rate, may still see significant impacts on farmland and the ecosystem due to its low elevation.

5.1.3 Cumulative Effects and Their Impacts

The compounding effects of sea level rise (SLR) and land subsidence not only lead to long-term land loss but also profoundly impact Shanghai’s infrastructure, ecological environment, and socioeconomic development.

(1) Infrastructure Vulnerabilities

The rise in relative sea level exposes coastal defenses (such as seawalls and tidal gates) to increased hydrostatic pressure, potentially exceeding their design thresholds and causing failure[30,31]. For instance, some of Shanghai’s tidal barriers were designed based on sea level standards from 50 years ago.If the relative sea level rises by more than 1.2 meters in the future, the original flood defense capability will be significantly compromised. Additionally, Land subsidence may cause structural deformation of infrastructure[32]. Especially in areas with deep soft soil foundations[33], such as certain metro lines in Pudong and Yangpu, which may face the risk of tunnel deformation due to foundation settlement

(2) Ecological Degradation

Saltwater intrusion intensifies the salinization of coastal wetlands, impairing the health of marine ecosystems. Furthermore, land subsidence alters the flow paths of river networks, degrading water quality[34]. Coastal wetlands and estuarine ecosystems, such as Chongming Dongtan Wetland—a vital stopover on the East Asian-Australasian Flyway for migratory birds[35]—face mounting pressures. However, as sea level rises and land subsides, the wetland

area shrinks, leading to a decline in ecosystem services[36,37].

(3) Socioeconomic Losses

Land inundation and infrastructure damage directly impact residents' lives and economic activities[38,39,40]. By 2100, economic losses in Shanghai's coastal industrial zones, logistics parks, and residential areas could reach hundreds of billions of yuan, with exorbitant relocation and reconstruction costs. Real estate markets may also suffer, as property values in low-lying coastal areas decline due to chronic flood risks[41]. Some residents may have to move, which can cause socioeconomic issues like housing shortages and employment pressures[42,43].

The combined effects of sea level rise and land subsidence significantly amplify Shanghai's flooding risk[44], especially during extreme events such as storm surges, when the disaster impacts are even more severe. Urban planning and disaster management need to comprehensively consider the long-term impacts of both, and adopt proactive adaptive measures to enhance the city's resilience to climate change.

5.2 Storm Surges Intensify the Destructive Power of Compound Disasters

In the context of global climate change, Shanghai faces multifaceted and complex disaster threats from sea level rise (SLR), land subsidence, and storm surges. Among these, storm surges—extreme weather events characterized by increasing frequency and intensity—significantly amplify disaster risks when compounded with SLR and subsidence[45].

5.2.1 Synergistic Effects of Storm Surges and SLR

Storm surges, triggered by intense weather systems such as typhoons and extratropical cyclones, are abrupt, high-intensity phenomena with widespread impacts[46]. Under global warming, rising sea surface temperatures and increased atmospheric moisture create favorable conditions for storm surge formation[47]. Climate change not only escalates the frequency and intensity of extreme weather events but also indirectly exacerbates storm surge threats by altering ocean circulation and atmospheric dynamics.

The synergistic effects of storm surges and SLR are particularly pronounced. SLR, a direct consequence of climate change, elevates the baseline water level for storm surges[48]. This means that even in the absence of storm surges, low-lying coastal areas will face heightened inundation risks. When storm surges occur, SLR further intensifies their destructive power, enabling tidal waters to more easily breach seawalls, expand inundation extents, and increase flooding depths[49]. Projections indicate that by 2100, Shanghai’s absolute SLR will reach 0.81 meters, while relative SLR (incorporating land subsidence) will rise to 1.24 meters. This upward trend in sea levels will undeniably magnify the threat of storm surges to Shanghai.

5.2.2 Compounding Impacts of Storm Surges and Land Subsidence

Land subsidence, driven by excessive groundwater extraction, compression of soft soil foundations, and rapid urbanization[50], poses another critical challenge for Shanghai. Subsidence lowers terrain elevation, rendering coastal areas more vulnerable to storm surges[51]. As shown in Table 2, cumulative subsidence in Shanghai is projected to reach 0.43 meters by 2100. This subsidence not only directly reduces land elevation but also destabilizes urban infrastructure, weakening its capacity to withstand storm surges.

When storm surges interact with SLR and subsidence, their destructive potential is significantly increased. Subsidence accelerates the rate of relative SLR, making low-lying coastal zones more susceptible to storm surge impacts[52]. Additionally, subsidence-lowered terrain reduces natural buffering against tidal intrusion, prolonging flood retention and expanding inundation areas. Furthermore, subsidence undermines the structural stability of coastal defenses (e.g., seawalls), diminishing their protective efficacy[53]. This compound interaction mechanism significantly elevates disaster risks for Shanghai during storm surge events.

5.2.3 Regularity Analysis of Storm Surge Impacts

Through an in-depth analysis of the data tables presented in this study, we further elucidate the regularity patterns of storm surge impacts. Temporally, as sea level rise (SLR) and land subsidence persist, storm surge-induced inundation risks for Shanghai will continue to escalate. As shown in Table 5, under Scenario 3 (SLR + subsidence + storm surges), the projected inundation area in Shanghai exhibits a year-by-year increasing trend from 2050 to 2100. This trend reflects the long-term and cumulative nature of interactions between storm surges, climate change, and land subsidence. Additionally, storm surge impacts demonstrate periodic characteristics, with frequency and intensity peaking during monsoon seasons and typhoon-active periods. Such periodicity implies heightened disaster risks for Shanghai during specific intervals, making it necessary to intensified weather monitoring, early warnings, and preventive measures to mitigate losses.

The interplay of storm surges, SLR, and land subsidence not only exacerbates disaster risks but also profoundly impacts Shanghai's socioeconomic development and ecological environment[54]. Inundation of low-lying coastal areas due to SLR and subsidence directly disrupts livelihoods and economic activities, leading to massive financial losses[55]. Prolonged flooding and expanded inundation zones may also cause irreversible damage to coastal wetlands and estuarine ecosystems, threatening biodiversity and ecological balance[56].

Under climate change, the destructive power of storm surges in Shanghai has intensified significantly. Their compound effects with SLR and subsidence continually elevate coastal

disaster risks. A thorough understanding of storm surge dynamics, compounding impacts, and regularity patterns is critical for formulating effective disaster prevention and mitigation strategies, ensuring Shanghai’s sustainable development.

5.3 Spatial heterogeneity significantly impacts composite risk distribution

Under global climate change, Shanghai faces complex disaster risks from sea level rise (SLR), land subsidence, and storm surges[57]. These hazards not only independently threaten urban safety and development but also interact to create a compounding "rising seas and sinking land" effect, significantly amplifying disaster risks. As a vast, topographically diverse, and socioeconomically concentrated international metropolis, Shanghai exhibits pronounced spatial heterogeneity in its exposure to these risks across different regions. This heterogeneity is reflected not only in natural geographic conditions but also in socioeconomic structures, disaster resilience capacities, and ecological conditions.

5.3.1 Spatial Heterogeneity in Natural Geographic Conditions

Shanghai is situated on the Yangtze River Delta alluvial plain, characterized by overall flat terrain but significant internal topographic variations. Eastern coastal areas, particularly Chongming Island and Pudong New Area, are low-lying and vulnerable to direct impacts from SLR and storm surges. In contrast, western and southern districts such as Qingpu and Songjiang, though also part of the plain, have relatively higher elevations and face lower immediate threats from SLR. Additionally, elevated regions like Dajinshan (103.4 meters) act as natural "safe islands," offering better resistance to floods and storm surges.

Land subsidence, another critical hazard, also displays marked spatial heterogeneity. Driven by excessive groundwater extraction, soft soil compression, and rapid urbanization, subsidence rates vary significantly across Shanghai[58]. Coastal zones like Pudong New Area and Baoshan District, with complex geology and intensive groundwater use, experience higher subsidence rates (e.g., 5–10 mm/year), exacerbating their disaster risks. Inland areas, with lower groundwater extraction and subsidence rates, remain comparatively safer.

5.3.2 Spatial Heterogeneity in Socioeconomic Activities

As China’s economic, financial, trade, and shipping hub, Shanghai’s socioeconomic activities are highly concentrated, with stark regional disparities in development levels and industrial structures. Coastal regions such as Pudong and Baoshan, benefiting from strategic locations and transportation networks, serve as vital industrial and logistics hubs. However, these areas face heightened disaster risks—floods or storm surges could cripple local economies.In contrast to coastal areas, inland regions in Shanghai have relatively lower economic development levels but more diversified industrial structures, resulting in smaller

impacts from disasters. For instance, Qingpu and Songjiang Districts, with their focus on agriculture and light industry, exhibit stronger resilience against natural disasters. These areas can also achieve sustainable economic development by fostering emerging industries such as eco-tourism and cultural creativity.

5.3.3 Spatial Heterogeneity in Disaster Resilience

Disaster resilience is a critical indicator of a city's capacity to respond to natural hazards. While Shanghai has achieved notable progress in disaster prevention and mitigation, significant disparities in resilience persist across its regions. Coastal areas, facing higher disaster risks, receive greater attention and investment from the government and stakeholders[59]. These zones prioritize infrastructure such as flood embankments and tidal gates, resulting in relatively stronger disaster resilience. In contrast, inland areas, with lower perceived risks, receive less focus and resources, leaving them underprepared for natural disasters. Moving forward, efforts in disaster management must emphasize regional balance and coordination, enhancing resilience in inland areas through targeted investments and capacity-building initiatives.

5.3.4 Spatial Heterogeneity in Ecological Conditions

Ecological conditions serve as a crucial indicator of a city's sustainable development potentia. Shanghai's ecological status varies markedly across regions. Coastal areas, subjected to long-term stressors like SLR, storm surges, and subsidence, exhibit fragile ecosystems. The wetlands, estuaries, and other ecosystems in these areas have suffered severe damage, leading to a decline in biodiversity and weakened ecosystem services. Inland regions, however, boast more stable ecosystems and higher biodiversity. By strengthening ecological conservation and restoration efforts, these areas can enhance ecosystem resilience, providing robust support for the city's sustainable development.

5.3.5 The comprehensive effect of spatial heterogeneity on the distribution of composite risk

The impact of spatial heterogeneity on compound risk distribution is multi - faceted. First, the spatial heterogeneity of natural geographical conditions determines the degree of vulnerability of different regions to hazards such as sea-level rise, land subsidence, and storm surges. Coastal areas, characterized by their low-lying topography and high rates of land subsidence, are prone to high disaster risks[60].Second, the spatial heterogeneity of socio - economic activities exacerbates the uneven distribution of disaster risks. As key economic hubs of Shanghai, coastal areas would suffer huge economic impacts if disasters struck. In contrast, inland areas, with relatively lower levels of economic development but diverse industrial structures, are less impacted by disasters.Furthermore, the spatial heterogeneity in disaster prevention and mitigation capabilities influences the response speed and effectiveness of different regions when dealing with disasters. Coastal areas, with relatively stronger disaster

prevention and mitigation capabilities, are better equipped to withstand disaster impacts.Lastly, the spatial heterogeneity of ecological and environmental conditions has a profound impact on urban sustainable development. Coastal areas should focus on ecological protection and restoration to enhance ecosystem resilience. Inland areas can support sustainable urban development by strengthening ecological conservation.

In summary, the impact of spatial heterogeneity on compound risk distribution is significant. Future disaster - prevention and mitigation work should consider the spatial heterogeneity of different regions, formulate differentiated strategies and measures, and achieve sustainable urban development and effective disaster - risk management.

6. Conclusions and Recommendations

6.1 Research Conclusions

6.1.1 Compound Effects Significantly Amplify Flood Risks

Shanghai's inundation risks are driven by the nonlinear compounding of sea level rise (SLR), land subsidence (LS), and storm surges (SS). Under IPCC AR6 projections and ARIMA modeling, the inundation area for SLR alone reaches 361.32 km² by 2100. With land subsidence added, this area increases to 460.97 km² (a 28% rise), while incorporating storm surges escalates it to 1,331.91 km². These findings emphasize that storm surges play a pivotal role in significantly amplifying the risks, especially in the context of cumulative subsidence. The interaction of relative SLR (SLR + LS) and storm surges disproportionately impacts low-lying regions such as Chongming District and Pudong New Area, where elevations are low and subsidence rates are high (10–20 mm/year in some zones). By 2100, Chongming District alone accounts for 725.28 km² of inundation (56% of the city's total).

6.1.2 Spatial Heterogeneity Shapes Regional Risk Distribution

The inundation risks in Shanghai demonstrate significant spatial variability, influenced by topography, subsidence rates, and land use patterns. Coastal lowlands (e.g., Chongming, Pudong, Baoshan) face far greater risks than inland areas due to lower elevations and higher subsidence. For instance, by 2100, Chongming's inundation area reaches 725.28 km², while Pudong's is 393.17 km². In contrast, inland districts like Jing'an and Huangpu remain largely unaffected.

The uneven spatial distribution of land subsidence exacerbates the vulnerability of coastal areas in Shanghai, especially in densely industrialized and residential zones like Pudong New District. Prolonged subsidence has resulted in lower land elevations, thereby increasing the pressure on flood defenses." In contrast, inland areas, with higher landforms and less subsidence, face smaller direct flood risks, but still need to guard against indirect impacts from extreme rainfall and storm surges, such as urban flooding.Therefore, Shanghai's disaster prevention

planning must adopt differentiated strategies based on regional characteristics to effectively tackle the challenges brought by spatial heterogeneity.

6.1.3 Long-Term Increase in Risks

Shanghai’s inundation risks escalate significantly over the long term (to 2100), particularly under combined SLR, subsidence, and storm surges. The differences between the scenario considering only SLR and the one combining SLR with LS are minimal in 2050 and 2070, suggesting that the short-term impacts of subsidence are limited. However, by 2100, SLR+LS inundation expands by 28% (460.97 km^2 vs. 361.32 km^2), highlighting the growing influence of cumulative subsidence. With storm surges added, inundation areas surge to 1,281.07 km^2 by 2050 and 1,331.91 km^2 by 2100—far exceeding the design capacity of existing defenses (e.g., seawalls at 3.0 meters). These trends underscore the urgent need to enhance infrastructure resilience and emergency preparedness.

6.2 Recommendations

6.2.1 Develop an Integrated Risk Management Framework

To address the multifaceted threats posed by climate change, Shanghai should establish an integrated risk management framework that combines climate adaptation, land subsidence control, and disaster emergency measures. This framework is designed to enhance urban resilience by leveraging data-driven approaches and fostering interagency collaboration.

First, the government should invest in a city-wide monitoring network to collect real-time data on sea-level rise, land subsidence, and storm surges, providing a scientific basis for decision - makers. Secondly, an interdepartmental coordination body should be established to integrate resources from meteorological, water conservancy, planning, and emergency management departments, breaking down information silos and ensuring policy consistency. Then, a risk assessment platform based on big data and AI should be developed. This platform utilizes predictive models to analyze potential flooding areas and disaster impacts, thereby optimizing emergency response plans. Additionally, city - wide risk assessments and actual combat drills should be conducted annually to test and improve the effectiveness of the framework. Through this comprehensive management framework, Shanghai can systematically address compound risks, enhance decision - making efficiency and emergency response capabilities, and maintain urban stability during extreme climate events.

6.2.2 Implement Spatially Differentiated Adaptation Strategies

Given the varying risk levels across Shanghai, differentiated policies should be implemented to optimize resource allocation. For high-risk areas like Chongming and Pudong, prioritize reinforcing sea defenses (by raising their heights to 7-8 meters), restoring wetlands,

and relocating residents to mitigate direct threats from sea-level rise and storm surges. Also, promote sponge city initiatives and upgrade drainage systems to enhance flood prevention. In low-risk areas such as Jing'an and Huangpu, concentrate on disaster education and community drills to boost residents' self - rescue capabilities and ensure swift responses during disasters. Additionally, the government should establish special funds for infrastructure upgrades in high-risk zones and offer tax incentives to encourage corporate participation in disaster prevention projects. This regional approach enables scientific resource distribution, effectively reducing citywide risks and ensuring Shanghai's overall safety.

6.2.3 Promote Technological Innovation and International Collaboration

Technological innovation is a critical means to enhance Shanghai's climate adaptation capacity. The government should encourage the utilization of remote sensing technology, high-precision digital elevation models (DEMs), and climate modeling technologies to precisely predict inundation extents and risk distribution, providing a scientific basis for urban planning. Concurrently, Shanghai should strengthen collaboration with global coastal cities (e.g., Tokyo, New York) to learn advanced practices in flood control engineering and risk management, while sharing technical resources. Additionally, dedicated research funds should be established to incentivize universities and enterprises to jointly develop adaptive technologies, such as salt-tolerant crops, green building materials, and smart flood control systems. These innovations will not only improve Shanghai's disaster resilience but also drive related industrial growth. Through international partnerships and technological R&D, Shanghai can position itself as a global leader in climate change adaptation, ensuring robust preparedness for future high-risk scenarios.

6.2.4 Strengthen Socioeconomic Resilience

To alleviate the economic and social impacts of climate disasters, Shanghai should adopt a comprehensive strategy encompassing promoting the development of green and low-carbon industries, relocating high-value facilities to lower-risk areas, and establishing robust disaster insurance mechanisms to reduce post-disaster recovery costs. In agricultural areas such as Chongming, promoting climate-resilient agricultural practices like flood-tolerant crops and eco-fisheries can ensure food security and ecological balance. Moreover, enhancing the social security system to provide post - disaster assistance for low - income groups can prevent disasters from worsening social inequalities.Upgrading infrastructure, improving climate monitoring, and refining early warning systems can boost preparedness and response capabilities. Additionally, fostering public awareness and encouraging community involvement in disaster preparedness and response efforts are crucial. By implementing these comprehensive measures, Shanghai can enhance its economic resilience, ensure the stability of residents' lives, and achieve sustainable development amidst climate challenges.

Authors Contributions

The first draft of the manuscript was written by BL. Data collection and analysis were performed by YxZ and Yx W. GqS contributed to the study conception and design.W Mark and Zg S have made revisions and improvements to the manuscript.

Acknowledgements

Thank Professor Sarah Rogers for reviewing and modifying the manuscript.

Funding

This research was supported by the National Social Science Fund of China （23CXW034） ;& Fundamental Research Funds for the Central Universities: Climate Migration Types and Risk Management in Coastal Areas. (grant number B230205032); &Postgraduate Research & Practice Innovation Program of Jiangsu Province: Climate Migration Types and Risk Management in Coastal Areas. (grant number 422003151); and The Key Research Project of the National Foundation of Social Science of China: Community Governance and Post-relocation Support in Cross District Resettlement [grant number 21&ZD183].

Data Availability Statement

The data and materials are available from the corresponding author on reasonable request.

Conflicts of Interest

The authors declare no conflict of interest.

Ethical approval

Ethical approval was not required as the study did not involve human participants.

Informed consent

This article does not contain any studies with human participants performed by any of the authors; therefore, obtaining consent was not required.

Reference

1. CAI Rong-Shuo,TAN Hong-Jian. Impacts and risks of accelerating sea level rise on low lying islands, coasts and communities. Climate Change Research, 2020, **16**(2): 163-171.

2. Sweet W, Horton R, Kopp R, et al. Sea level rise. 2017.

3. IPCC. Climate Change 2023: Synthesis Report: Contribution of Working Groups I, II and III to the Sixth Assessment Report of the Intergovernmental Panel on Climate Change [R]. Geneva: IPCC, 2023: 35-115. doi: 10.59327/IPCC/AR6-9789291691647.

4. Azevedo de Almeida B, Mostafavi A. Resilience of infrastructure systems to sea-level

rise in coastal areas: Impacts, adaptation measures, and implementation challenges. Sustainability, 2016, **8**(11): 1115.

5. Ruan X, Sun H, Shou W, et al. The impact of climate change and urbanization on compound flood risks in coastal areas: a comprehensive review of methods. Applied Sciences, 2024, **14**(21): 10019.

6. Pal I, Kumar A, Mukhopadhyay A. Risks to coastal critical infrastructure from climate change. Annual Review of Environment and Resources, 2023, **48**(1): 681-712.

7. Zurui Ao et al.,A national-scale assessment of land subsidence in China's major cities.Science,2024,**384**:301-306.doi:10.1126/science.adl4366.

8. Fang J, Nicholls R J, Brown S, et al. Benefits of subsidence control for coastal flooding in China. Nature Communications, 2022, **13**(1): 6946.

9. FANG Jia-Yi, YIN Jie, SHI Xian-Wu, FANG Jian, DU Shi-Qiang, LIU Min. A review of compound flood hazard research in coastal areas. Climate Change Research, 2021, **17**(3): 317-328.doi:10.12006/j.issn.1673-1719.2020.210.

10. Church J A, Clark P U, Cazenave A, et al. Sea-level rise by 2100. Science, 2013, **342**(6165): 1445-1445.

11. Cazenave A, Cozannet G L. Sea level rise and its coastal impacts. Earth's Future, 2014, **2**(2): 15-34.

12. Frederikse T, Landerer F, Caron L, et al. The causes of sea-level rise since 1900. Nature, 2020, **584**(7821): 393-397.

13. Nicholls R J, Cazenave A. Sea-level rise and its impact on coastal zones. science, 2010, **328**(5985): 1517-1520.

14. Rahmstorf S. Rising hazard of storm-surge flooding. Proceedings of the National Academy of Sciences, 2017, **114**(45): 11806-11808.

15. Yin J, Jonkman S, Lin N, et al. Flood risks in sinking delta cities: time for a reevaluation?. Earth's Future, 2020, **8**(8): e2020EF001614.

16. Hwang C, Yang Y, Kao R, et al. Time-varying land subsidence detected by radar altimetry: California, Taiwan and north China. Scientific reports, 2016, **6**(1): 28160.

17. Abidin H Z, Andreas H, Gumilar I, et al. Land subsidence of Jakarta (Indonesia) and its relation with urban development. Natural hazards, 2011, **59**: 1753-1771.

18. Erkens G, Bucx T, Dam R, et al. Sinking coastal cities. Proceedings of the International Association of Hydrological Sciences, 2015, **372**(372): 189-198.

19. Wang T, Yang Z. The nonlinear response of storm surge to sea-level rise: a modeling approach. Journal of Coastal Research, 2019, **35**(2): 287-294.

20. Kirezci E, Young I R, Ranasinghe R, et al. Projections of global-scale extreme sea levels and resulting episodic coastal flooding over the 21st Century. Scientific reports, 2020, **10**(1): 11629.

21. Gregory J M, Griffies S M, Hughes C W, et al. Concepts and terminology for sea level: Mean, variability and change, both local and global[J]. Surveys in Geophysics, 2019, **40**: 1251-1289.

22. Chatfield C. The analysis of time series: theory and practice. Springer, 2013.

23. Nicholls R J, Lincke D, Hinkel J, et al. A global analysis of subsidence, relative sea-level change and coastal flood exposure. Nature Climate Change, 2021, **11**(4): 338-342.

24. Khan A A. TEMPORARY REMOVAL: Why would sea-level rise for global warming and polar ice-melt?. 2019.HANG Yecheng, HU Jingjiang, et al. Spatial and temporal distribution characteristics

25. ZHANG Chunshan, Z and formation conditions of geological disasters in China. Quaternary Sciences, 2000, **20**(6): 559-566.

26. Galloway D L, Bawden G W, Leake S A, et al. Land subsidence hazards. Landslide and land subsidence hazards to pipelines: US Geological Survey Open-File Report, 2008, 1164.

27. Cantelon J A, Kurylyk B L. Storm surge, seawater flooding, and sea-level rise paradoxically drive fresh surface water expansion. Environmental Research Letters, 2024, **19**(12): 124038.

28. Wang J, Gao W, Xu S, et al. Evaluation of the combined risk of sea level rise, land subsidence, and storm surges on the coastal areas of Shanghai, China. Climatic change, 2012, **115**: 537-558.

29. Wheater H, Evans E. Land use, water management and future flood risk. Land use policy, 2009, **26**: S251-S264.

30. Azevedo de Almeida B, Mostafavi A. Resilience of infrastructure systems to sea-level rise in coastal areas: Impacts, adaptation measures, and implementation challenges. Sustainability, 2016, **8**(11): 1115.

31. Arns A, Dangendorf S, Jensen J, et al. Sea-level rise induced amplification of coastal protection design heights. Scientific reports, 2017, **7**(1): 40171.

32. LIN Hui, MA Peifeng, WANG Weixi. Introduction to the spaceborne MT-InSAR method for monitoring the health of urban infrastructure. 2017.

33. WU Huaina, GU Weihua, SHEN Shuilong. Assessment of the impact of regional land subsidence on the long-term settlement of Shanghai subway tunnels. Shanghai Land & Resources, 2017, **38**(2).

34. Bagheri-Gavkosh M, Hosseini S M, Ataie-Ashtiani B, et al. Land subsidence: A global challenge. Science of The Total Environment, 2021, **778**: 146193.

35. Liu B, Gao H, Wang J, et al. Migratory Connectivity of Zhejiang, with a Critical Stopover in East Asian-Australasian Flyway, Based on Recovery Data. Animals, 2024, **14**(16): 2404.

36. Hinkel J, Schuerch M, French J, et al. Sea-level rise risk and adaptation in estuaries//Climate change and estuaries. CRC Press, 2023: 581-602.

37. van de Pol M, Bailey L D, Frauendorf M, et al. Sea-level rise causes shorebird population collapse before habitats drown. Nature Climate Change, 2024, **14**(8): 839-844.

38. Hammond M J, Chen A S, Djordjević S, et al. Urban flood impact assessment: A state-of-the-art review. Urban Water Journal, 2015, **12**(1): 14-29.

39. Cortés Arbués I, Chatzivasileiadis T, Ivanova O, et al. Distribution of economic damages due to climate-driven sea-level rise across European regions and sectors. Scientific Reports, 2024, **14**(1): 1-15.

40. Oddo P C, Lee B S, Garner G G, et al. Deep uncertainties in sea-level rise and storm surge projections: Implications for coastal flood risk management. Risk Analysis, 2020, **40**(1): 153-168.

41. Tyndall J. Sea level rise and home prices: evidence from Long Island. The Journal of Real Estate Finance and Economics, 2023, **67**(4): 579-605.

42. Hauer M E, Fussell E, Mueller V, et al. Sea-level rise and human migration. Nature Reviews Earth & Environment, 2020, **1**(1): 28-39.

43. Lincke D, Hinkel J. Coastal migration due to 21st century sea-level rise. Earth's Future, 2021, **9**(5): e2020EF001965.

44. Ao Z, Hu X, Tao S, et al. A national-scale assessment of land subsidence in China’s major cities. Science, 2024, **384**(6693): 301-306.

45. XU Yijian. Development strategy of China’s coastal cities in response to climate change. Climate Change Research, 2020, **16**(1): 88.

46. Gordon G A, Young R R. Natural hazards: hurricanes, cyclones, and typhoons. Encyclopedia of security and emergency management, 2021: 676-683.

47. Michener W K, Blood E R, Bildstein K L, et al. Climate change, hurricanes and tropical storms, and rising sea level in coastal wetlands. Ecological applications, 1997, **7**(3): 770-801.

48. CAI Rongshuo, TAN Hongjian. Impacts and risks of accelerated sea-level rise on low-lying islands, coastal areas, and society. Climate Change Research, 2020, **16**(2): 163.

49. van de Wal R S W, Melet A, Bellafiore D, et al. Sea Level Rise in Europe: impacts and consequences. State of the Planet Discussions, 2023, 2023: 1-65.

50. Bagheri-Gavkosh M, Hosseini S M, Ataie-Ashtiani B, et al. Land subsidence: A global challenge. Science of The Total Environment, 2021, **778**: 146193.

51. YANG Huating. Sea-level rise along the Chinese coast and coastal disasters. Quaternary Sciences, 1999, **19**(5): 456-465.

52. Johnston J, Cassalho F, Miesse T, et al. Projecting the effects of land subsidence and sea level rise on storm surge flooding in Coastal North Carolina. Scientific Reports, 2021, **11**(1): 21679.

53. Wang Hanmei, Jiao Xun. Control Strategy of Land Subsidence in Shanghai Under Sea-Level Rise. Climate Change Research, 2015, **11**(4): 256-262.

54. Xu H, Hou X, Pan S, et al. Socioeconomic impacts from coastal flooding in the 21st century China's coastal zone: A coupling analysis between coastal flood risk and socioeconomic development. Science of the Total Environment, 2024, **917**: 170187.

55. Hsiao S C, Fu H S, Wu H L, et al. Impact assessment of sea level rise-induced high tide flooding and socioeconomic losses in a highly vulnerable coastal region. Journal of Hydrology: Regional Studies, 2024, **55**: 101921.

56. Bao J, Gao S. Wetland utilization and adaptation practice of a coastal megacity: a case study of Chongming Island, Shanghai, China. Frontiers in Environmental Science, 2021, **9**: 627963.

57. Shan X, Yin J, Wang J. Risk assessment of shanghai extreme flooding under the land use change scenario. Natural Hazards, 2022, **110**(2): 1039-1060.

58. ZHANG Jianwei, HU Ke, YUE Wei, et al. Ground subsidence in Shanghai urban area and its response to groundwater extraction and recharge. Resources and Environment in the Yangtze River Basin, 2016, **25**(4): 567-572.

59. LI Tianxing. Research progress on sustainable development indicator systems at home and abroad. Journal of Ecology and Environment, 2013, **22**(6): 1085-1092.

60. Zhao Q, Pepe A, Devlin A, et al. Impact of sea-level-rise and human activities in coastal regions: an overview. Journal of Geodesy and Geoinformation Science, 2021, **4**(1): 124.
