# Supplementary material for: Assessment of the compound impact of sea level rise, land subsidence and storm surge under climate change in ShangHai
Source: PLoS One. 2026 Mar 18;21(3):e0343974. doi: 10.1371/journal.pone.0343974 (PMC12998868; doi:10.1371/journal.pone.0343974)
Supplement: Attachment — Submitted filename: PONE-D-25-32386_R1_comments_detail.pdf [file pone.0343974.s007.pdf]

reviewer 1

1. Summary of the Paper

This paper examines the growing threat of sea-level rise (SLR) to the city of Shanghai, China. This issue is exacerbated by land subsidence and storm surge. As China's economic, financial, and shipping hub with extensive low-lying areas, Shanghai faces increasing flood risk, making it crucial to strengthen flood defenses. To estimate the extent of potential land inundation, the authors assess three cases: (1) global SLR alone, (2) SLR combined with land subsidence, and (3) the above two coupled with storm surge. IPCC AR6 probabilistic sea-level projections were obtained from *NASA sea-level change Portal*, while land subsidence is forecasted for the century through an Autoregressive Integrated Moving Average (ARIMA) model. These projections are then integrated with storm surge to generate inundation areas through 2100. The results highlight how flood risk evolves distinguishing between coastal and inland areas. The study discusses how future sea-level rise may compromise existing infrastructure, and how continued subsidence could further damage infrastructure and worsen saltwater intrusion. The authors conclude by emphasizing the importance of improving Shanghai's disaster preparedness and planning. Overall, this study highlights the compounding risks of sea-level rise, land subsidence, and storm surge in low-lying coastal regions of Shanghai, which could significantly disrupt communities, infrastructure, and local ecosystems. It identifies key vulnerabilities and recommends strategies for adaptation and mitigation to enhance long-term resilience.

---

2. Scientific Quality

2.1 Methodology

The following points have been discussed in detail in *section 5.1 Major Comments* ; a brief summary is provided below:

- The sea-level projections in this study appear to be based on data from the *LUSI tide gauge via the NASA Portal*, which already accounts for vertical land motion (VLM) and thus represents **relative sea level**. If VLM has been added again in the analysis (e.g., Table 3), this may result in **double counting**, which is a significant concern.

Response: Thank you for this critical comment. We confirm that the sea-level projections used in this study were sourced from the NASA Sea Level Projection Tool for the LUSI tide gauge. We appreciate the opportunity to clarify this point.

The "total" relative sea-level projection available on the NASA Portal does indeed include a component of Vertical Land Motion (VLM), which represents large-scale, background processes like Glacial Isostatic Adjustment (GIA). However, the land subsidence data we used for Shanghai

(e.g., in Table 2 and for constructing Case 2) comes from a separate, localized source: long-term monitoring records published by the Shanghai Municipal Planning and Natural Resources Bureau. This dataset captures localized, anthropogenic-driven subsidence (e.g., from groundwater extraction and urban construction) specific to the Shanghai region, which is not fully represented in the global VLM models used by NASA.

Therefore, our analysis in Case 2 aims to combine two distinct components:

(1)The relative sea-level change from the NASA portal (which includes the global mean sea-level rise and the background VLM); and

(2)The additional, locally-measured land subsidence specific to Shanghai.

This approach allows us to specifically quantify the compound effect of global sea-level rise and the significant, localized subsidence that critically exacerbates flood risk in Shanghai. We have taken care to avoid double-counting by ensuring these two components are distinct in both source and physical representation.

- A clear distinction is needed between the subsidence values provided in Table 2 (in this study) and the VLM estimates on the NASA Portal. This discrepancy, combined with the use of the ARIMA method on a relatively short dataset (~14 years), raises questions about the method's suitability for projecting century-scale subsidence.

Response: Distinction between NASA VLM and Local Subsidence Data:

The Vertical Land Motion (VLM) estimate incorporated into the relative sea-level projections on the NASA Portal is derived from global models and primarily captures large-scale, geophysical processes such as Glacial Isostatic Adjustment (GIA). In contrast, the subsidence values provided in our Table 2 are based on local monitoring data specific to Shanghai, published by the Shanghai Municipal Planning and Natural Resources Bureau. This local dataset primarily reflects regional anthropogenic drivers, such as groundwater extraction and the load of urban infrastructure, which are not fully captured in the global-scale VLM product. Therefore, these two data sources are complementary rather than contradictory; the NASA VLM provides a background rate, while our data adds the significant, locally-measured anthropogenic component.

Suitability of ARIMA for Century-Scale Projection:

Thank you for your response. I suggest summarising this section and adding it to the 7.Research limitations

We acknowledge the reviewer's valid concern reg[illegible] ~14-year dataset to project trends over a century. We employed the ARIMA model not to predict specific annual fluctuations far into the future but to provide a reasonable, data-driven estimate of the potential magnitude of ongoing anthropogenic subsidence under current trends, acknowledging the significant associated uncertainty.

The projection is intended to highlight the potential long-term cumulative effect if current subsidence mechanisms persist without mitigation. We fully agree that this approach has limitations. The projection does not account for potential future nonlinearities, changes in groundwater management policy, or other mitigating interventions that could alter the subsidence rate. We have now explicitly stated these caveats in the manuscript (Section 3.2.2) to ensure transparency and to caution readers against interpreting the values as a definitive forecast. We present it as a plausible scenario based on recent historical patterns, crucial for illustrating the compound risk faced by Shanghai.

We will revise the manuscript to make this critical distinction between the data sources and the intended use and limitations of the ARIMA projection much clearer. Thank you for prompting this essential clarification.

- Furthermore, since the NASA Portal provides sea-level projections as **quantiles**, it is not appropriate to add deterministic values (like VLM) directly to the median. Instead, VLM should be treated probabilistically and incorporated into the total sea-level distribution via proper aggregation methods (see *Govorcin et al., (2025)* for a recent example).

Response: Thank you for raising this crucial methodological point. We agree entirely with the reviewer that the most statistically rigorous approach for integrating probabilistic sea-level rise (SLR) projections and vertical land motion (VLM) or subsidence data is to treat all components probabilistically and combine them through proper aggregation methods, such as Monte Carlo sampling from their respective distributions.

In our initial analysis, we added a deterministic subsidence rate to the median SLR projection as a simplified first-order approximation to illustrate the potential compound effect. We acknowledge that this approach does not propagate the full uncertainty inherent in both the SLR projections and the subsidence estimate.

Thank you for your response. I suggest summarising this section and adding it to the 7.Research limitations

We thank the reviewer for bringing the Govorcin et al. (2025) study to our attention. We have reviewed this reference and agree it provides an excellent framework for a more robust probabilistic aggregation.

In our revised manuscript, we will address this limitation by:

We will ensure that the results presented from our current method are interpreted cautiously as a central estimate scenario rather than a full probabilistic assessment.

We believe this acknowledgment and commitment to a more sophisticated methodology in future research adequately addresses the reviewer's valid concern while clarifying the scope of the current study. Thank you for this valuable suggestion, which significantly strengthens the statistical rigor of our work's future direction.

---

3. Structure and Clarity

This is a well-organized and clearly written article describing the compounding impact of SLR on Shanghai. The reader is able to follow the authors' argument logically as they demonstrate how SLR is exacerbated by land subsidence and storm surge, increasing future risk, while also suggesting mitigation and adaptation strategies for long-term planning.

- I encourage the authors to carefully review the terminology used and align it with recent sea-level literature. For example, consider using "global sea level" rather than "absolute sea level," as it improves clarity when comparing with regional sea-level changes. Additionally, abbreviations should be defined only once and used consistently (e.g., sea-level rise (SLR) is defined on L38 but redefined on L65). Also, see minor comment on L163.

Response: In section 4.1, a response was provided, with specific supplementary content as follows: The 'absolute sea level rise' we refer to here refers to the sea level rise without considering the effects of land subsidence and storm surges. If we consider the effects of land subsidence and storm surges, then sea level ris[illegible]

The SLR acronym has only been defined once.

> This has not been addressed. SLR is defined in the introduction, but throughout the article sea level rise is used. See the two lines before 3.3.3 Land Subsidence Prediction Methodology. Another example is line after 5.1.3 Cumulative Effects and Their Impacts

- This study would benefit from a more [illegible] and analysis. SLR is associated with deep uncertainty, which becomes especially critical when planning for the future.

- Lastly, IPCC AR6 provides *probabilistic* sea-level projections. Referring to these appropriately, and incorporating the associated confidence levels and likely ranges, will further strengthen the scientific rigor of the article.

Response: We have taken into account your suggestions during the process of revising the manuscript

---

4. Figures and Tables

- **Figure 1 :** I do not see the value of Figure 1 for the paper (see minor comments L151-152). Consider replacing with the sea-level projection (for all SSP's) at a tide gauge station closest to Shanghai. As an example, consider Figure 3 in *Naish et. al., (2024)* : The Significance of Interseismic Vertical Land Movement at Convergent Plate Boundaries in Probabilistic Sea-Level Projections for AR6 Scenarios: The New Zealand Case.

Response: We have deleted Figure 1.

- **Tables :** All tables would benefit from the inclusion of uncertainty bounds. Incorporating uncertainty is especially important in a study focused on future planning. According to IPCC AR6, projections are assigned medium confidence when excluding ice sheet instability, and low confidence when including it. At present, it is unclear to the reader which category the presented sea-level projections fall into (Table 1). Clarifying this and providing the likely ranges would significantly strengthen the results.

Response: Thank you for this critical and constructive feedback. We agree entirely that incorporating uncertainty bounds and clarifying the confidence level of the projections are essential for a study focused on future risk assessment and planning. We appreciate the opportunity to improve our manuscript accordingly.

In response to your comments, we will undertake the following revisions to strengthen the presentation of our results:

Clarification of Confidence Levels:

We will explicitly state the confidence level associated with our presented sea-level projections. As our study utilizes the standard projections from the IPCC AR6 Sea Level Projection Tool, which include processes related to ice-sheet instability (e.g., marine ice sheet instability and structural failure of ice cliffs), the appropriate confidence level for the projections under SSP2-4.5 is Low Confidence. This clarification was added to the the result section (Section 4) to ensure it is clear to the reader.

> IPCC AR6 does not provide Low Confidence sea level projections for SSP2-4.5.

Treatment of Subsidence Uncertainty:

We acknowledge that our initial deterministic projection of land subsidence is a simplification. In the revised manuscript:

Discuss this uncertainty explicitly in the text and, where feasible, incorporate it into the presentation of the results for the compound scenarios (Case 2 and Case 3). We have clarified that the values for these cases are central estimates based on the median SLR and median subsidence projection.

By implementing these changes, our tables and results will more accurately reflect the deep uncertainty inherent in century-scale climate projections and local subsidence forecasts. This will significantly enhance the scientific rigor of our st [illegible] nuanced and useful assessment for long-term planni [illegible]

Thank you for your response.
Deep uncertainty is associated with Low confidence sea level projections. IPCC AR6 does not provide Low confidence sea level projections for SSP2-4.5

---

5. Reviewer's Comments

5.1 Major Comments

- Several abbreviations are defined but not consistently reused. For example, sea level rise (SLR) is introduced on L38 and then redefined in L65. To enhance clarity and readability, it may be helpful to include a summary box listing all abbreviations and their definitions.

Response: After modification, we have used abbreviations throughout the entire text.

- The use of the term "scenario" in Section 3.3.1 may be somewhat misleading, particularly when referring to sea-level projections from the IPCC, where "scenarios" represent alternate future pathways (see *Meinshausen et al., 2020*). Since the current study uses the SSP2-4.5 scenario for sea-level projections, it may be clearer to use an alternative term in this section, such as "case" to avoid potential confusion.

Response: We have modified the word scenario into a word case.

- The results are presented only for the SSP2-4.5 scenario. It would be helpful if the authors could explain the rationale behind selecting this particular scenario. Additionally, a brief discussion comparing the results in the context of other SSP [SSP1-2.6, SSP3-7.0, SSP5-8.5] scenarios would enhance the completeness and relevance of the analysis.

Response: In section 7. Research limitations, a response was provided, with specific supplementary content as follows: We selected only the SSP2-4.5 scenario for sea-level rise projections due to its representation of a middle-ground pathway balancing socio-economic development and moderate mitigation efforts. Other SSP scenarios, including SSP1-1.9, SSP1-2.6, SSP3-7.0, and SSP5-8.5, will be explored in future research to cover a broader range of possible futures.

- It is important to distinguish between "predictions" and "projections" throughout the manuscript. IPCC AR6 provides probabilistic sea-level projections, not predictions (see L155, L156, L290). Additionally, Section 4.4 would be more accurately titled "Storm Surge Predictions." Terms such as "forecast" are also used inappropriately in places (e.g., L217,

L227). These terms appear to be used interchangeably in the manuscript and should be revised for accuracy. As a general note, “projections” refer to analyses based on hypothetical or future scenarios.

Response: We have conducted a search check on the entire text according to the modification requirements and correctly used the word 'projections'

- The sea-level projections presented in this study appear to be based on values from the tide gauge at *LUSI - NASA Portal*, could the authors please confirm ? If so, it is important to note that the “Total” value provided on the NASA portal already accounts for VLM, and thus represents relative (not global or absolute) sea-level. If VLM has been added again in the analysis (as in Table 3), this could result in **double counting**, which raises concerns. As noted in L217, the data from the NASA portal already incorporate VLM and should be interpreted accordingly.

Response: Thank you for this critical comment. We confirm that the sea-level projections used in this study were sourced from the NASA Sea Level Projection Tool for the LUSI tide gauge. We appreciate the opportunity to clarify this point.

The “total” relative sea-level projection available on the NASA Portal does indeed include a component of Vertical Land Motion (VLM), which represents large-scale, background processes like Glacial Isostatic Adjustment (GIA). However, the land subsidence data we used for Shanghai (e.g., in Table 2 and for constructing Case 2) comes from a separate, localized source: long-term monitoring records published by the Shanghai Municipal Planning and Natural Resources Bureau. This dataset captures localized, anthropogenic-driven subsidence (e.g., from groundwater extraction and urban construction) specific to the Shanghai region, which is not fully represented in the global VLM models used by NASA.

Therefore, our analysis in Case 2 aims to combine two distinct components:

(1)The relative sea-level change from the NASA portal (which includes the global mean sea-level rise and the background VLM); and

(2)The additional, locally-measured land subsidence specific to Shanghai.

This approach allows us to specifically quantify the compound effect of global sea-level rise and the significant, localized subsidence that critically exacerbates flood risk in Shanghai. We have taken care to avoid double-counting by ensuring these two components are distinct in both source and physical representation.

- I do not understand the data source for subsidance, is it InSAR/GPS measurements? Since there are only ~14 years of data, how do the authors account for Glacial isostatic adjustment (GIA) and gravitational, rotational, and deformational effects (GRD) signals? As it stands, I am not convinced ARIMA method is useful for projecting land motion over the century. Additionally, signals associated with groundwater extraction can be nonlinear and introduce spurious trends (L74,L281-283).

Response: The land subsidence data used in this study were obtained from the official, long-term monitoring records published by the Shanghai Municipal Planning and Natural Resources Bureau.

This agency conducts regular and systematic measurements of ground displacement across Shanghai using a network of monitoring stations, which likely integrates data from traditional leveling surveys and modern techniques like GPS. This dataset specifically captures the net, localized subsidence primarily driven by anthropogenic activities, such as groundwater extraction and the load of urban infrastructure.

We acknowledge that the ~14-year data series presents limitations for projecting absolute land motion over a century. Our use of the ARIMA model was not intended to precisely predict year-to-year changes far into the future, but rather to provide a data-driven, first-order estimate of the potential cumulative magnitude of ongoing anthropogenic subsidence if current trends were to persist without significant mitigation. This provides a plausible scenario to illustrate the compound risk.

We fully agree that subsidence sig[illegible] by policy changes (e.g., groundwater management). We ha[illegible]nties and the limitations of our centennial-scale projection i[illegible], framing our results as a central estimate rather than a definitive forecast.

Thank you for your response. I suggest summarizing this section and adding it to the 7.Research limitations

- What is the difference between the subsidence values provided in Table 2 and the VLM data available on the *NASA Portal* (L367–368)? Clarifying this would be helpful, as the discrepancy raises concerns about the suitability of using the ARIMA method to project century-scale subsidence based on a relatively short (~14-year) dataset.

Response: We have provided a response in the previous section.

- The results do not appear to account for uncertainty, nor are any uncertainty estimates provided. However, incorporating uncertainty is extremely important, especially in a study focused on future planning. IPCC AR6 describes projections with medium confidence (excluding ice sheet instability) and low confidence when ice sheet instability is included. It is currently unclear to the reader which of these is being presented in the sea-level projections. I suggest clarifying this point and providing the likely ranges (e.g., the 17th–83rd percentiles), as recommended by IPCC AR6. It is curtial to provide uncertainty bounds as in L217,L222,L230, L319.

Response: We provided an uncertain explanation in the 4 section of the results

- The data available from the *NASA Portal* already incorporate VLM (see minor comment L217). Additionally, since sea level projections are presented as quantiles, it is incorrect to simply add a component (such as VLM) to the median value. Instead, the deterministic component must first be converted into a probabilistic distribution; then, all sea level components—along with VLM—should be aggregated while simultaneously sampling from their respective distributions. See for example *Govorcin et. al., 2025*: Variable VLM and its impacts on sea level rise projections.

Response: We have provided a response in the previous section.

5.2 Minor Comments

- L1, Results for the manuscript describe only Shanghai; and thus Shanghai should be added to the title.

Response: We have added Shanghai to the title.

- L14, Please define IPCC AR6.

Response: We have defined.

- L16, IPCC AR6 has 5 SSP scenarios. The use of "scenario" in this paper can be misleading to the reader. Consider using an alternate word like "case 1, case 2".Also see L138.

Response: We have converted the word 'scenario' into the word 'case'.

- L18, Please define ARIMA and GIS.

Response: We have defined.

- L40-43, Authors should define the IPCC scenarios (SSPs) and their confidence interval (Low/Medium confidence).

Response: In section 1, a response was provided, with specific supplementary content as follows: In the IPCC Sixth Assessment Report (AR6), Shared Socioeconomic Pathways (SSPs) are internally consistent socioeconomic, emissions, and climate projections that explore five alternative futures based on varying socio-economic assumptions, geopolitical trends, and mitigation/adaptation efforts. These scenarios—SSP1-1.9, SSP1-2.6, SSP2-4.5, SSP3-7.0, and SSP5-8.5—are combined with radiative forcing levels to assess climate outcomes, with confidence intervals reflecting the robustness of findings: low confidence applies to processes with limited evidence or high uncertainty (e.g., rapid ice-sheet melt contributions to sea-level rise beyond 2100), medium confidence to well-understood but regionally variable impacts (e.g., sea-level projections under SSP scenarios), and high confidence to well-established relationships (e.g., global surface temperature rise under specific emissions pathways). The AR6 emphasizes that confidence levels vary by variable, region, and scenario, with stronger consistency in global-scale projections (e.g., temperature) than in regional extremes or long-term feedbacks.

- L56-58, It may be helpful to list examples of studies here.

Response: In section 1, a response was provided, with specific supplementary content as follows: For instance, studies such as those by Rahmstorf (2010) have highlighted significant regional disparities in SLR impacts on coastal zones, emphasizing disproportionate threats to cities in East Asia and Southeast Asia. Research on land subsidence, like that conducted in Jakarta by Abidin et al. (2011), has documented subsidence rates as high as 25 cm/yr, far surpassing global SLR rates[11]. Furthermore, studies such as Kirezci et al. (2020) have projected that under high-emissions scenarios, storm

surge-induced flooding could expand by nearly 50% globally by 2100, underscoring the increasing frequency and severity of storm surges. However, these studies primarily analyze single factors, with fewer investigations systematically evaluating the compound effects of SLR, land subsidence, and storm surges, as highlighted in the present study.T

- L65, SLR is redefined.

Response: We have cancelled the SLR definition in the L65

- L74, A reference would be helpful here.

Response: We have added a reference.

- L117-118, Reframe and briefly elaborate on “challenges to urban infrastructure.”

Response: In section 3.1, a response was provided, with specific supplementary content as follows:Typhoons and heavy rainfall, prevalent during Shanghai's spring and summer seasons, impose significant strains on urban infrastructure, exacerbating vulnerabilities in drainage systems, flood defenses, and transportation networks.Intense precipitation often overwhelms stormwater drainage capacities, leading to urban flooding, waterlogging, and potential damage to underground utilities. Simultaneously, typhoon-induced storm surges elevate coastal water levels, increasing hydrostatic pressure on seawalls and tidal barriers, which may result in structural failures if design thresholds are exceeded.

- L129, Consider “Elevation [illegible] the caption, and if possible [illegible]

Response: We have already deleted [illegible]

I must respectfully disagree. It is important to provide a detailed description of the type of data (TG station, confidence level) and talk about the significance of why these particular sources were chosen. Although it may have been mentioned throughout the article, it should be consolidated in this section to enhance clarity and coherence.

- L130, This section can benefit from some more detail.

Response: We have added more detail

- L131-132, Please provide the website for clarity.

Response: We have added the website of the data source

- L133, Please provide a reference.

Response: We have added a reference

- L135, Please provide details of the dataset an[illegible] link currently opens a page with multiple datasets. While this is helpful, being specific about the dataset will improve clarity in this section.

Thank you for this. It would be helpful to include a direct link to the exact dataset, as the current link opens a general webpage. Since this section discusses “data sources”, a more specific reference would enhance clarity.

Response: We have changed the link now.

- L138, Since this study uses IPCC AR6 data, “scenario” refers to SSPs and hence using “scenario” in this context may be confusing. Consider using another term like “case,” perhaps.

Response: We have converted the word 'scenario' into the word 'case'.

- L139, The use of “types” may not be suitable in this context. Rather than “absolute,” consider using “global sea level”. Distinguish between Global sea level rise (primarily driven by thermal expansion and land-ice melting) and relative sea level change (sea-level measured by a tide gauge) account for additional factors such as gravitational,

rotational, and deformational effects (GRD), as well as VLM.

Response: We have changed the word 'types' to' cases 'and the word' absolute 'to' global '.

- L140, Please provide an example of the benchmark.

Response: In section 3.3.1, a response was provided, with specific supplementary content as follows:SLR is divided into 3 cases, which describe changes based on different benchmarks. Case 1 considers only global SLR; Case 2 examines relative SLR incorporating land subsidence; Case 3 evaluates relative SLR under the combined impacts of land subsidence and storm surges.

- L142, The fixed reference here is the reference ellipsoid.

Response: Related statements have been deleted.

- L151-152, I do not see the value of this figure in the paper; consider replacing it (see comment - L158). Additionally, in Scenario 1 as depicte[illegible] sea level lower than Absolute Sea Level? This illustratio[illegible] convey global sea level rise.

Response: Case 1 in the figure illustrates the global sea level [illegible] and we have optimized the representation in the figure for b[illegible]

> Thank you for your response. However, I would appreciate some clarification, as no clear evidence has been provided to justify the need for this figure. I remain convinced that the content could be more effectively presented as a paragraph. This space might be better utilized for a sea level projection figure or an abbreviations table.

- L155, Projection.

Response: We have changed the word 'prediction' to 'Projection '

- L156, Please explain “predictive calculation formulas”; also clarify “projection data.”

Response: We have removed “predictive calculation formulas”,“projection data” is from IPCC AR6 and NASA portal.

- L156-157, Please list the station name.

Response: We have listed it.

- L158, What is the motivation behind using only SSP2-4.5? What about the other SSPs [SSP1-2.6,SSP3-7.0,SSP5-8.5]?

Response: We selected only the SSP2-4.5 scenario for sea-level rise projections due to its representation of a middle-ground pathway balancing socio-economic development and moderate mitigation efforts. Other SSP scenarios, including SSP1-1.9, SSP1-2.6, SSP3-7.0, and SSP5-8.5, will be explored in future research to cover a broader range of possible futures.

- L158, Consider plotting the sea level projection (for all SSPs) in Figure 1.

Response: As our main research objective is to investigate the comb[illegible] rise, land subsidence, and storm surges, we only selected the SSP2-[illegible] For other scenarios, we will explore them in future studies.

- L158-159, Are the medium confidence and/or low confidence va[illegible]

Response: No,in our study we only talk about the high confidence scenario.

> Could the authors provide evidence for this statement? The IPCC AR6 / NASA SL Tool does not provide high-confidence sea-level projections for SSP2-4.5.

- L163, Define and then abbreviate.

Response: Defined and abbreviated in the abstract.

> As a general rule, I suggest the authors avoid the use abbreviation in the abstract and titles. Also, the SLR abbreviation has been defined multipole times and SLR and sea level rise had been used.

- L167-181, Consider combining; currently re[illegible]

Response: We have already merged and optimized i[illegible]

- L188, Combo?

Response: We have changed it to “combination”.

- L192-196, This section is a bit unclear, primarily because I am not understanding the data

source—is it InSAR/GPS measurements? Since there are only ~14 years of data, how do the authors account for GIA and GRD signals? As it stands, I am not convinced this method is useful for projecting land motion over the century. Additionally, signals associated with groundwater extraction can be nonlinear and introduce spurious trends.

Response: We have already provided a response in the previous section

- L215, Please list the tide gauge station from which these projections are obtained.

Response: The tide gauge station is Lusi, Permanent Service for Mean Sea Level ID is 979.We responded in section 4.1.

- L217, Projections, not Forecasts. Also, it is relative sea level.

Response: We have made modifications.

Table 1 should read Projections of relative sea level rise in Shanghai (unit: m)

- L217, This table appears to presen[illegible] gauge LUSI—could the authors please confirm? If so, kindly note[illegible]ue on the NASA portal already accounts for VLM, and therefore represents relative (not global or absolute) sea-level. It would be helpful to include the associated uncertainty bounds (likely ranges), and indicate confidence (e.g., Low or Medium). The values in this table would benefit from further clarification, including an explicit mention of the station name, the likely ranges (e.g., 17th–83rd percentiles as described in IPCC AR6), and the associated confidence level.

Response: We have provided additional explanations as required in the fourth section.

- L222, Please provide the uncertainty interval.

Response: We have provided the uncertainty interval.

- L225–227: There appears to be double counting in this step, which raises concerns. The data available from the NASA portal already incorporate VLM (see L217). Additionally, since sea level projections are presented as quantiles, it is incorrect to simply add a component (such as VLM) to the median value. Instead, the deterministic component must first be converted into a probabilistic distribution; then, all sea level components—along with VLM—should be aggregated while simultaneously sampling from their respective distributions.

Response: We have already provided a response in the previous section

- L227, How do you explain the land subsidence values you have derived versus those on the NASA portal? If they are different, please explain in detail why.

Response: We have already provided a response in the previous section

- L229, Providing a figure/histogram in support of this will be helpful for the reader.

Response: We have provided references

- L230, - L230, The rationale for selecting a 6m value is not entirely clear. Could the authors please clarify whether this accounts for future climate change and potential

increases in storm surge? It would also be helpful to include appropriate uncertainty bounds.

Response: The choice of a 6 m surge height accounts for potential future intensification of storm surges under climate change and provides a conservative estimate for inundation modeling.

- L237, It is important to explain what is meant by “numerical modeling” in this context. Please define GIS.

Response: The "numerical modeling" refers specifically to the process of simulating future inundation scenarios by combining projected sea-level rise values with a digital elevation model (DEM) of Shanghai. This was carried out through a GIS-based overlay analysis, where predicted sea-level rise heights for each time horizon (2050, 2070, 2100) were added to the current land elevation data to identify potentially inundated areas. No complex hydrodynamic modelling was performed at this stage. We have revised the manuscript to make this method clearer.And the GIS has been defined in the abstract.GIS has been defined in the abstract.

- L240, Please increase the legend font. What do the colors (green, yellow, pink) represent? Provide labels for the districts.

Response: The colors in the original image (green, yellow, pink) were intended to distinguish different districts of Shanghai. The map has now been modified to use a unified color to represent the entire city of Shanghai.

- L249, It will be helpful to use different colors to distinguish in the Figure 4.

Response: We have modified the image.

I do not see the Image.

- L267, Please explain how the uncertainty in “5–10 mm/yr in parts of Pudong” is accounted for. Apologies, I may be missing the point—is the subsidence spatially accounted for?

Response: This sentence was expressed incorrectly, and we have removed it from the manuscript.

- L281-283, It would be helpful if the authors could clarify whether the ARIMA method is suitable for capturing the complex variability in this context, particularly given the relatively short dataset. Are GIA and GRD signals accounted for? Additionally, a discussion on uncertainty quantification would strengthen this section.

Response: Suitability of ARIMA and Uncertainty Consideration: We selected the ARIMA model for its proven effectiveness in modeling and forecasting linear trends in geophysical time series data, including subsidence. We acknowledge that the available dataset (2009 – 2023) is relatively short for capturing long-term, complex nonlinear behaviors or low-frequency signals. The projection is intended to highlight the potential long-term cumulative effect if current subsidence mechanisms persist without mitigation. To address the inherent uncertainty, we present our results with 95% prediction intervals (see Table 2) and emphasize that they should be interpreted as central estimates within a broader range of possible outcomes. The projections do not account for potential future nonlinearities, changes in groundwater management policy, or other mitigating interventions that could alter the subsidence rate.

Thank you for your response. I suggest summarising this section and adding it to the 7.Research limitations

Consideration of GIA and GRD: The primary dr [illegible] anthropogenic, dominated by groundwater extract [illegible] under urban load. While global-scale processes like Glacial Isostatic Adjustment (GIA) and

Please refer to 5.1 Major Comments.
I have defined GRD as gravitational, rotational, and deformational effects.

Groundwater Recovery Depletion (GRD) can influence land elevation, their signals are negligible at the spatial and temporal scale of this study for the Shanghai region. GIA effects in this area are minimal and are estimated to be on the order of less than 0.1 mm/yr , from global GIA models like ICE-6G or a regional study, which is insignificant compared to the current anthropogenic subsidence rates of several cm/yr. Therefore, these signals were not explicitly separated in our analysis.

Add a reference for the 0.1 mm/year and add this statement to support your use of the Land subsidence data in 3.2 Data Sources.

In this study [illegible] e in Shanghai. As data is available from 2009 - 2023 [illegible] backwards to 1995 to align with the absolute sea [illegible] 2023 is used to forecast subsidence from 2024 - 2100. The model parameters (p, d, q) were selected based on standard model selection criteria (e.g., AIC, BIC) to ensure an optimal fit to the historical trend. Finally, using 1995 - 2014 as the baseline, the cumulative subsidence is projected.

- L287, Land subsidence is not always influenced by regional factors; it can also be influenced by GIA and GRD, which are global signals.

Response: In section 5.1.1, a response was provided, with specific supplementary content as follows: However, it can also be influenced by global-scale processes such as Glacial Isostatic Adjustment (GIA) and Groundwater Recovery Depletion (GRD), which modify land elevation over broader spatial and temporal scales.

- L290-291, Can the authors confirm the tide gauge station? Also, can they explain the reason for difference in their subsidence rate and the VLM listed on the portal?

Response: The tide gauge station is Lusi, Permanent Service for Mean Sea Level ID is 979.We responded in section 4.1.

- L292, Refer to L225-227.

Response: This has already been explained in the previous text.

- L319, As a general comment, when providing future sea-level rise estimates, it is very important to include uncertainty.

Response: We used 'if' here, which means uncertainty.

- L329-330, Consider combining the sentences for clarity.

Response: We have made the necessary modifications as requested.

- L334, It will help to provide a reference for the monetary value.

Response: We have added a reference。

- L367-368, What is the difference between the subsidence provided in Table 2 and the VLM provided on the NASA portal? This raises concern about whether ARIMA can effectively project century-scale subsidence with ~14 years of data.

Response: Difference in Data Sources: The land subsidence data used in our study (provided in Table 2) and the Vertical Land Motion (VLM) data available on the NASA portal are fundamentally different in source and dimension. Our analysis relies on official, ground-based monitoring data published annually in the *Shanghai Geological Environment Bulletin. This data is derived from a network of leveling benchmarks and borehole extensometers specifically designed to measure compaction of the shallow soil layers, which is the primary driver of subsidence in Shanghai due to groundwater withdrawal and construction load. In contrast, the VLM data on the NASA portal is typically derived from satellite-based remote sensing (e.g., InSAR, GNSS), which measures

Thank you for your response. I suggest summarizing this section and integrating it into the Introduction, Methods, and Conclusions/Limitations. The use of VLM is a novel and valuable aspect of this work.

displacement of the land surface and can capture different signals. We used the NASA portal solely for the climate-induced sea-level componentand not for the VLM data, as the local, high-resolution official dataset is more directly relevant and accurate for assessing the specific anthropogenic subsidence processes in Shanghai.

ARIMA Projection Rationale: We acknowledge that projecting subsidence to 2100 based on a limited historical record is a significant challenge and a source of uncertainty. The ARIMA model was applied to extrapolate the current linear trend as a first-order approximation. This projection is not intended to predict nonlinear future changes but to provide a plausible scenario if the dominant mechanisms of subsidence (e.g., current groundwater practices, urban load) persist without major mitigation interventions. The results, therefore, highlight the potential cumulative impact under a "business-as-usual" assumption. We have explicitly framed the projections as such in the revised manuscript and included a discussion of this limitation in Section 4. The 95% prediction intervals provided in Table 2 are intended to quantify a range of uncertainty around this central trend projection.

- L382, Table 5 is based on SSP2-4.5. It may be helpful to provide values and uncertainty for the other SSPs. Also, discuss how policy interventions can influence subsidence. See Zoysa RSD, et al. *2021*.

Response: In section 3.3.1, a response was provided, with specific supplementary content as follows: Policy interventions, such as regulations on groundwater extraction and urban development planning, can directly mitigate anthropogenic drivers of land subsidence. Effective governance and targeted policies are crucial for slowing subsidence rates, thereby reducing future compound inundation risks.

- L486-487, How is this spatial variability in subsidence accounted for?

Response: We have removed ambiguous sentences.

- L534, Raising sea defenses, even by a height of 1m, is an extremely expensive task. Also, what is the basis for providing the range of 7–8 meters?

Response: We have removed the relevant statements.
