# Supplementary material for: Assessment of the compound impact of sea level rise, land subsidence and storm surge under climate change in ShangHai
Source: PLoS One. 2026 Mar 18;21(3):e0343974. doi: 10.1371/journal.pone.0343974 (PMC12998868; doi:10.1371/journal.pone.0343974)
Supplement: Attachment — Submitted filename: review1.pdf [file pone.0343974.s009.pdf]

R1 response to author Comments

Title

Assessment of the compound impact of sea level rise, land subsidence and storm surge under climate change.

Authors
Bing Liang, Guoqing Shi, Yuexi Wu, Yuxuan Zhu, Mark Wang, Zhonggen Sun

Recommendation

- ☐ Accept as is
- ☒ Accept with minor revisions
- ☐ Major revision required
- ☐ Reject OR
  - ☐ Reject & Resubmit (Editors descission)

---

1. Summary of the Comments

While the authors did a great job addressing my previous comments, there are still *two* outstanding areas that need to be fixed.

1. **Results** section: Sea-level projection **Confidence**
   - The projections are **medium confidence**. Look at your screen-shot, the first line explicitly mentions it. **Only** SSP1-2.6 and SSP5-8.5 have low-confidence projections.
   - **Change this in** Section 4: Results, **change Low Confidence** to medium confidence.

2. It is `Projections` not predictions.
   - Make this change in `3.2 Data Sources`.
   - Since ~~Working Group I of~~ the IPCC AR6 has already ~~made predictions~~ produced regional sea-level projections ~~the rise in global sea level~~, this study adopts these ~~predicted~~ projected values for the following research. The IPCC AR6 ~~predicted~~ provides projections of sea level ~~on SLR~~ for five Shared Socioeconomic Pathways (SSP) including SSP1-1.9,SSP1-2.6, SSP2-4.5, SSP3-7.0 and SSP5-8.5 based on the average sea level from 1995 to 2014 ~~and are divided into two different scenarios: the SSP scenario and the Future~~

~~Global Warming Levels scenario.The SSP is a new generation of scenario combinations constructed by the IPCC for comprehensive analysis of future climate change.~~ These scenarios depict various different development patterns of future economic and social systems, reflecting the correlation between economic and social development and the challenges of mitigating and adapting to climate change. They are the core foundation for conducting climate change impact assessments and climate policy formulation. ~~These include SSP1-1.9,SSP1-2.6, SSP2-4.5, SSP3-7.0, SSP5-8.5, and other scenarios.~~ To highlight the research theme, which is the combined impact of SLR, land subsidence, and storm surges on coastal cities, and to reduce computational workload, we ~~selected the sea-level rise prediction~~ use sea-level projection data under the ~~medium carbon~~ emission scenario ~~of~~ SSP2-4.5 for analysis. The ~~prediction~~ projected data was downloaded from the NASA Sea Level Change Portal.

The name of the tide gauge station we selected is "LUSI", which is located in China. The station is situated in Lvsigang Town, Rudong County, Jiangsu Province, and is one of the important tide gauge stations along the Yellow Sea coast, frequently used for research on sea level changes in China's coastal waters. The query website is: https://sealevel.nasa.gov/ipcc-ar6-sea-level-projection-tool?psmsl_id=979&data_layer=scenario.
