# Supplementary material for: Assessment of the compound impact of sea level rise, land subsidence and storm surge under climate change in ShangHai
Source: PLoS One. 2026 Mar 18;21(3):e0343974. doi: 10.1371/journal.pone.0343974 (PMC12998868; doi:10.1371/journal.pone.0343974)
Supplement: Attachment — Submitted filename: Response_to_Reviewers_auresp_4.pdf [file pone.0343974.s011.pdf]

R1 response to Reviewers' Comments

Reference 33 is a retracted article and you have to change this. Also, please check the reference style through out. Why is there another uncited reference in 33 (HANG Yecheng et al). Are other references correctly cited (as per the numbering)?

33. Khan A A. TEMPORARY REMOVAL: Why would sea-level rise for global warming
and polar ice-melt?. 2019.HANG Yecheng, HU Jingjiang, et al. Spatial and temporal
distribution characteristics

Response： We have replaced the 33rd reference with a new one: Larour E, Seroussi H, Adhikari S, et al. Slowdown in Antarctic mass loss from solid Earth and sea-level feedbacks[J]. Science, 2019, 364(6444): eaav7908.

The coding of other cited references is also correct.
