# Supplementary material for: Assessment of the compound impact of sea level rise, land subsidence and storm surge under climate change in ShangHai
Source: PLoS One. 2026 Mar 18;21(3):e0343974. doi: 10.1371/journal.pone.0343974 (PMC12998868; doi:10.1371/journal.pone.0343974)
Supplement: Attachment — Submitted filename: reviewer 1.docx [file pone.0343974.s002.docx]

# Paper Review

## Title

Assessment of the compound impact of sea level rise, land subsidence and storm surge under climate change.

**Authors**
Bing Liang, Guoqing Shi, Yuexi Wu, Yuxuan Zhu, Mark Wang, Zhonggen Sun

### Recommendation

- Accept as is
- Accept with minor revisions
- Major revision required
- Reject

## 1. Summary of the Paper

This paper examines the growing threat of sea-level rise (SLR) to the city of Shanghai, China. This issue is exacerbated by land subsidence and storm surge. As China’s economic, financial, and shipping hub with extensive low-lying areas, Shanghai faces increasing flood risk, making it crucial to strengthen flood defenses. To estimate the extent of potential land inundation, the authors assess three cases: (1) global SLR alone, (2) SLR combined with land subsidence, and (3) the above two coupled with storm surge. IPCC AR6 probabilistic sea-level projections were obtained from [NASA sea-level change Portal](https://sealevel.nasa.gov/ipcc-ar6-sea-level-projection-tool), while land subsidence is forecasted for the century through an Autoregressive Integrated Moving Average (ARIMA) model. These projections are then integrated with storm surge to generate inundation areas through 2100. The results highlight how flood risk evolves distinguishing between coastal and inland areas. The study discusses how future sea-level rise may compromise existing infrastructure, and how continued subsidence could further damage infrastructure and worsen saltwater intrusion. The authors conclude by emphasizing the importance of improving Shanghai’s disaster preparedness and planning. Overall, this study highlights the compounding risks of sea-level rise, land subsidence, and storm surge in low-lying coastal regions of Shanghai, which could significantly disrupt communities, infrastructure, and local ecosystems. It identifies key vulnerabilities and recommends strategies for adaptation and mitigation to enhance long-term resilience.

## 2. Scientific Quality

### 2.1 Methodology

The following points have been discussed in detail in section 5.1 Major Comments ; a brief summary is provided below:

- The sea-level projections in this study appear to be based on data from the [LUSI tide gauge via the NASA Portal](https://sealevel.nasa.gov/ipcc-ar6-sea-level-projection-tool?psmsl_id=979&data_layer=scenario), which already accounts for vertical land motion (VLM) and thus represents **relative sea level**. If VLM has been added again in the analysis (e.g., Table 3), this may result in **double counting**, which is a significant concern.
- A clear distinction is needed between the subsidence values provided in Table 2 (in this study) and the VLM estimates on the NASA Portal. This discrepancy, combined with the use of the ARIMA method on a relatively short dataset (~14 years), raises questions about the method’s suitability for projecting century-scale subsidence.
- Furthermore, since the NASA Portal provides sea-level projections as **quantiles**, it is not appropriate to add deterministic values (like VLM) directly to the median. Instead, VLM should be treated probabilistically and incorporated into the total sea-level distribution via proper aggregation methods (see [Govorcin et al., (2025)](https://doi.org/10.1126/sciadv.ads8163) for a recent example).

## 3. Structure and Clarity

This is a well-organized and clearly written article describing the compounding impact of SLR on Shanghai. The reader is able to follow the authors’ argument logically as they demonstrate how SLR is exacerbated by land subsidence and storm surge, increasing future risk, while also suggesting mitigation and adaptation strategies for long-term planning.

- I encourage the authors to carefully review the terminology used and align it with recent sea-level literature. For example, consider using “global sea level” rather than “absolute sea level,” as it improves clarity when comparing with regional sea-level changes. Additionally, abbreviations should be defined only once and used consistently (e.g., sea-level rise (SLR) is defined on L38 but redefined on L65). Also, see minor comment on L163.
- This study would benefit from a more detailed treatment of uncertainty quantification and analysis. SLR is associated with deep uncertainty, which becomes especially critical when planning for the future.
- Lastly, IPCC AR6 provides *probabilistic* sea-level projections. Referring to these appropriately, and incorporating the associated confidence levels and likely ranges, will further strengthen the scientific rigor of the article.

## 4. Figures and Tables

- **Figure 1 :** I do not see the value of Figure 1 for the paper (see minor comments L151-152). Consider replacing with the sea-level projection (for all SSP’s) at a tide gauge station closest to Shanghai. As an example, consider Figure 3 in [Naish et. al., (2024)](https://doi.org/10.1029/2023EF004165) : The Significance of Interseismic Vertical Land Movement at Convergent Plate Boundaries in Probabilistic Sea-Level Projections for AR6 Scenarios: The New Zealand Case.
- **Tables :** All tables would benefit from the inclusion of uncertainty bounds. Incorporating uncertainty is especially important in a study focused on future planning. According to IPCC AR6, projections are assigned medium confidence when excluding ice sheet instability, and low confidence when including it. At present, it is unclear to the reader which category the presented sea-level projections fall into (Table 1). Clarifying this and providing the likely ranges would significantly strengthen the results.

## 5. Reviewer’s Comments

### 5.1 Major Comments

- Several abbreviations are defined but not consistently reused. For example, sea level rise (SLR) is introduced on L38 and then redefined in L65. To enhance clarity and readability, it may be helpful to include a summary box listing all abbreviations and their definitions.
- The use of the term “scenario” in Section 3.3.1 may be somewhat misleading, particularly when referring to sea-level projections from the IPCC, where “scenarios” represent alternate future pathways (see [Meinshausen et al., 2020](https://doi.org/10.5194/gmd-13-3571-2020)). Since the current study uses the SSP2-4.5 scenario for sea-level projections, it may be clearer to use an alternative term in this section, such as “case” to avoid potential confusion.
- The results are presented only for the SSP2-4.5 scenario. It would be helpful if the authors could explain the rationale behind selecting this particular scenario. Additionally, a brief discussion comparing the results in the context of other SSP [SSP1-2.6, SSP3-7.0, SSP5-8.5] scenarios would enhance the completeness and relevance of the analysis.
- It is important to distinguish between “predictions” and “projections” throughout the manuscript. IPCC AR6 provides probabilistic sea-level projections, not predictions (see L155, L156, L290). Additionally, Section 4.4 would be more accurately titled “Storm Surge Predictions.” Terms such as “forecast” are also used inappropriately in places (e.g., L217, L227). These terms appear to be used interchangeably in the manuscript and should be revised for accuracy. As a general note, “projections” refer to analyses based on hypothetical or future scenarios.
- The sea-level projections presented in this study appear to be based on values from the tide gauge at [LUSI - NASA Portal](https://sealevel.nasa.gov/ipcc-ar6-sea-level-projection-tool?psmsl_id=979&data_layer=scenario), could the authors please confirm ? If so, it is important to note that the “Total” value provided on the NASA portal already accounts for VLM, and thus represents relative (not global or absolute) sea-level. If VLM has been added again in the analysis (as in Table 3), this could result in **double counting**, which raises concerns. As noted in L217, the data from the NASA portal already incorporate VLM and should be interpreted accordingly.
- I do not understand the data source for subsidance, is it InSAR/GPS measurements? Since there are only ~14 years of data, how do the authors account for Glacial isostatic adjustment (GIA) and gravitational, rotational, and deformational effects (GRD) signals? As it stands, I am not convinced ARIMA method is useful for projecting land motion over the century. Additionally, signals associated with groundwater extraction can be nonlinear and introduce spurious trends (L74,L281-283).
- What is the difference between the subsidence values provided in Table 2 and the VLM data available on the [NASA Portal](https://sealevel.nasa.gov/ipcc-ar6-sea-level-projection-tool?psmsl_id=979&data_layer=scenario) (L367–368)? Clarifying this would be helpful, as the discrepancy raises concerns about the suitability of using the ARIMA method to project century-scale subsidence based on a relatively short (~14-year) dataset.
- The results do not appear to account for uncertainty, nor are any uncertainty estimates provided. However, incorporating uncertainty is extremely important, especially in a study focused on future planning. IPCC AR6 describes projections with medium confidence (excluding ice sheet instability) and low confidence when ice sheet instability is included. It is currently unclear to the reader which of these is being presented in the sea-level projections. I suggest clarifying this point and providing the likely ranges (e.g., the 17th–83rd percentiles), as recommended by IPCC AR6. It is curtial to provide uncertainty bounds as in L217,L222,L230, L319.
- The data available from the [NASA Portal](https://sealevel.nasa.gov/ipcc-ar6-sea-level-projection-tool?psmsl_id=979&data_layer=scenario) already incorporate VLM (see minor comment L217). Additionally, since sea level projections are presented as quantiles, it is incorrect to simply add a component (such as VLM) to the median value. Instead, the deterministic component must first be converted into a probabilistic distribution; then, all sea level components—along with VLM—should be aggregated while simultaneously sampling from their respective distributions. See for example [Govorcin et. al., 2025](https://doi.org/10.1126/sciadv.ads8163): Variable VLM and its impacts on sea level rise projections.

### 5.2 Minor Comments

- L1, Results for the manuscript describe only Shanghai; and thus Shanghai should be added to the title.
- L14, Please define IPCC AR6.
- L16, IPCC AR6 has 5 SSP scenarios. The use of “scenario” in this paper can be misleading to the reader. Consider using an alternate word like “case 1, case 2”.Also see L138.
- L18, Please define ARIMA and GIS.
- L40-43, Authors should define the IPCC scenarios (SSPs) and their confidence interval (Low/Medium confidence).
- L56-58, It may be helpful to list examples of studies here.
- L65, SLR is redefined.
- L74, A reference would be helpful here.
- L117-118, Reframe and briefly elaborate on “challenges to urban infrastructure.”
- L129, Consider “Elevation map of Shanghai.” Please describe what the colors mean in the caption, and if possible, increase the legend font size.
- L130, This section can benefit from some more detail.
- L131-132, Please provide the website for clarity.
- L133, Please provide a reference.
- L135, Please provide details of the dataset and the direct link to the data. The link currently opens a page with multiple datasets. While this is helpful, being specific about the dataset will improve clarity in this section.
- L138, Since this study uses IPCC AR6 data, “scenario” refers to SSPs and hence using “scenario” in this context may be confusing. Consider using another term like “case,” perhaps.
- L139, The use of “types” may not be suitable in this context. Rather than “absolute,” consider using “global sea level”. Distinguish between Global sea level rise (primarily driven by thermal expansion and land-ice melting) and relative sea level change (sea-level measured by a tide gauge) account for additional factors such as gravitational, rotational, and deformational effects (GRD), as well as VLM.
- L140, Please provide an example of the benchmark.
- L142, The fixed reference here is the reference ellipsoid.
- L151-152, I do not see the value of this figure in the paper; consider replacing it (see comment - L158). Additionally, in Scenario 1 as depicted in the figure, why is the mean sea level lower than Absolute Sea Level? This illustration in Scenario 1 does not seem to convey global sea level rise.
- L155, Projection.
- L156, Please explain “predictive calculation formulas”; also clarify “projection data.”
- L156-157, Please list the station name.
- L158, What is the motivation behind using only SSP2-4.5? What about the other SSPs [SSP1-2.6,SSP3-7.0,SSP5-8.5]?
- L158, Consider plotting the sea level projection (for all SSPs) in Figure 1.
- L158-159, Are the medium confidence and/or low confidence values included?
- L163, Define and then abbreviate.
- L167-181, Consider combining; currently reads with a lot of repetition.
- L188, Combo?
- L192-196, This section is a bit unclear, primarily because I am not understanding the data source—is it InSAR/GPS measurements? Since there are only ~14 years of data, how do the authors account for GIA and GRD signals? As it stands, I am not convinced this method is useful for projecting land motion over the century. Additionally, signals associated with groundwater extraction can be nonlinear and introduce spurious trends.
- L215, Please list the tide gauge station from which these projections are obtained.
- L217, Projections, not Forecasts. Also, it is relative sea level.
- L217, This table appears to present values from the tide gauge LUSI—could the authors please confirm? If so, kindly note that the “Total” value on the NASA portal already accounts for VLM, and therefore represents relative (not global or absolute) sea-level. It would be helpful to include the associated uncertainty bounds (likely ranges), and indicate confidence (e.g., Low or Medium). The values in this table would benefit from further clarification, including an explicit mention of the station name, the likely ranges (e.g., 17th–83rd percentiles as described in IPCC AR6), and the associated confidence level.
- L222, Please provide the uncertainty interval.
- L225–227: There appears to be double counting in this step, which raises concerns. The data available from the NASA portal already incorporate VLM (see L217). Additionally, since sea level projections are presented as quantiles, it is incorrect to simply add a component (such as VLM) to the median value. Instead, the deterministic component must first be converted into a probabilistic distribution; then, all sea level components—along with VLM—should be aggregated while simultaneously sampling from their respective distributions.
- L227, How do you explain the land subsidence values you have derived versus those on the NASA portal? If they are different, please explain in detail why.
- L229, Providing a figure/histogram in support of this will be helpful for the reader.
- L230, - L230, The rationale for selecting a 6m value is not entirely clear. Could the authors please clarify whether this accounts for future climate change and potential increases in storm surge? It would also be helpful to include appropriate uncertainty bounds.
- L237, It is important to explain what is meant by “numerical modeling” in this context. Please define GIS.
- L240, Please increase the legend font. What do the colors (green, yellow, pink) represent? Provide labels for the districts.
- L249, It will be helpful to use different colors to distinguish in the Figure 4.
- L267, Please explain how the uncertainty in “5–10 mm/yr in parts of Pudong” is accounted for. Apologies, I may be missing the point—is the subsidence spatially accounted for?
- L281-283, It would be helpful if the authors could clarify whether the ARIMA method is suitable for capturing the complex variability in this context, particularly given the relatively short dataset. Are GIA and GRD signals accounted for? Additionally, a discussion on uncertainty quantification would strengthen this section.
- L287, Land subsidence is not always influenced by regional factors; it can also be influenced by GIA and GRD, which are global signals.
- L290-291, Can the authors confirm the tide gauge station? Also, can they explain the reason for difference in their subsidence rate and the VLM listed on the portal?
- L292, Refer to L225-227.
- L319, As a general comment, when providing future sea-level rise estimates, it is very important to include uncertainty.
- L329-330, Consider combining the sentences for clarity.
- L334, It will help to provide a reference for the monetary value.
- L367-368, What is the difference between the subsidence provided in Table 2 and the VLM provided on the NASA portal? This raises concern about whether ARIMA can effectively project century-scale subsidence with ~14 years of data.
- L382, Table 5 is based on SSP2-4.5. It may be helpful to provide values and uncertainty for the other SSPs. Also, discuss how policy interventions can influence subsidence. See Zoysa RSD, et al. [2021](https://doi.org/10.1371/journal.pone.0250208).
- L486-487, How is this spatial variability in subsidence accounted for?
- L534, Raising sea defenses, even by a height of 1m, is an extremely expensive task. Also, what is the basis for providing the range of 7–8 meters?
