# Supplementary material for: Assessment of the compound impact of sea level rise, land subsidence and storm surge under climate change in ShangHai
Source: PLoS One. 2026 Mar 18;21(3):e0343974. doi: 10.1371/journal.pone.0343974 (PMC12998868; doi:10.1371/journal.pone.0343974)
Supplement: Attachment — Submitted filename: Reviewer 2.docx [file pone.0343974.s003.docx]

**Reviewer Comments**:

This manuscript presents an integrated assessment of sea level rise (SLR), land subsidence (LS), and storm surge (SS) impacts on Shanghai under climate change scenarios. This paper addresses the compound impacts of sea level rise (SLR), land subsidence (LS), and storm surges (SS) on Shanghai, a major global coastal megacity. Using IPCC AR6 projections, long-term land subsidence monitoring, and historical storm surge data, the authors construct three scenarios (SLR alone, SLR + LS, SLR + LS + SS) to project inundation risks for 2050, 2070, and 2100. Methods include ARIMA time-series modeling, GIS-based spatial analysis, and numerical simulation. Results indicate that storm surges significantly amplify inundation areas from 361.32 km² under SLR alone to 1,331.91 km² when combined with LS and SS by 2100. Spatial heterogeneity is emphasized, with Chongming District and Pudong New Area identified as high-risk zones. The paper concludes with adaptation recommendations, including integrated risk management frameworks, differentiated regional strategies, technological innovation, and socioeconomic resilience building.

**Areas for Improvement**

**Grammar and Style** – The manuscript contains several minor grammatical errors and awkward phrasings (e.g., inconsistent tense usage, missing articles, overly long sentences). A careful proofreading pass or professional language editing is recommended.

**Citations in Study Area Section** – The “Study Area” description currently lacks in-text references for geographic and factual details. These should be supported with authoritative sources.

**Introduction Expansion** – While the introduction covers SLR, LS, and SS, it would benefit from more recent and detailed global and regional statistics on sea level rise (e.g., IPCC AR6 scenario ranges, regional SLR acceleration rates, tide gauge records for Shanghai). This will strengthen the scientific context and underscore the urgency of the study.

**In the review section**, the literature review should more explicitly differentiate the novelty of this approach from prior compound-impact modelling studies in other regions.

**Integration of Socioeconomic Dimensions**

The paper is methodologically rigorous in physical hazard assessment but gives limited treatment to socioeconomic exposure and vulnerability. Integrating population density, critical infrastructure, and economic value data into spatial analysis would enhance decision-making applicability.

**Formatting**: Correct minor typographical issues (e.g., “centur[3]” → “century [3]”; missing space before citations).

**Terminology Clarification**: For a multidisciplinary audience, briefly define terms such as “relative sea level rise” and “ARIMA modeling” in non-technical language when they are first mentioned.

**Recommendations Section**: The recommendations are comprehensive but could be more concise to avoid repetition.
