# Supplementary material for: Assessment of the compound impact of sea level rise, land subsidence and storm surge under climate change in ShangHai
Source: PLoS One. 2026 Mar 18;21(3):e0343974. doi: 10.1371/journal.pone.0343974 (PMC12998868; doi:10.1371/journal.pone.0343974)
Supplement: Attachment — Submitted filename: review1_Comments.docx [file pone.0343974.s005.docx]

# R1 response to author Comments

## Title

Assessment of the compound impact of sea level rise, land subsidence and storm surge under climate change.

**Authors**
Bing Liang, Guoqing Shi, Yuexi Wu, Yuxuan Zhu, Mark Wang, Zhonggen Sun

### Recommendation

- Accept as is
- Accept with minor revisions
- Major revision required
- Reject OR
  - Reject & Resubmit (Editors descission)

## 1. Summary of the Comments

While the authors did a great job responding to the comments, there are still areas where the manuscript needs significant improvement. I recommend rejection of this manuscript in its current form, as several major issues remain unresolved despite the authors’ efforts to respond to prior comments. These include (and are discussed in detail below) inconsistencies in figure references (e.g., Figure 4 is still cited despite the deletion of figures), abbreviations, unresolved concerns regarding data uncertainty treatment, and overall clarity and presentation. Collectively, these issues indicate that the manuscript requires substantial further revision before it can meet publication standards. However, if the editor deems it appropriate, the authors could be invited to resubmit after fully addressing these major revisions.

1. The grammar of the article requires further polishing (there are missing spaces).
   - I remain unconvinced that the use of abbreviations has been fully addressed. For instance, while sea level change is abbreviated as SLR, both SLR and sea level rise are used, which creates confusion.
   - Also, two figures were deleted, but I want to flag that Figure 4 is still mentioned in the text, which suggests the manuscript needs a more thorough review.
   - This is one of the reasons I am currently recommending rejection

- This study is not yet adequate for use in planning, as it still does not provide uncertainty information needed for decision-making. Although the study is framed as a first attempt, the research limitations (Section 7) should be explicitly and clearly stated.
  - While the authors attempt to present uncertainty estimates, Tables 3, 4, and 5 do not incorporate uncertainty into the analysis. If confidence levels exist for the VLM data, I strongly recommend conducting a Monte Carlo simulation to meaningfully integrate sea-level data with VLM data. If this cannot be done, the authors must clearly state that the results should be interpreted with caution, particularly by those using the findings for planning purposes, due to the absence of quantified uncertainty.
  - This is one of the reasons I am currently recommending rejection, as it was raised in the first round of review but has not been fully addressed.
- low-confidence sea level projections for SSP2-4.5.
  - I urge the authors to revisit the data sources carefully. In their response, the authors stated: *“By implementing these changes, our tables and results will more accurately reflect the deep uncertainty inherent in century-scale climate projections and local subsidence forecasts.”* However, deep uncertainty is typically associated with low-confidence sea level projections.
  - Since IPCC AR6 does not provide low-confidence sea level projections for SSP2-4.5, this suggests either a misunderstanding or that different datasets are being referenced.
  - The authors also claim to use high-confidence scenario (response to comment L158–159); however, I am not familiar with such sea level projection categories, and these do not appear to be available via the NASA Sea Level Change Portal.
  - It is extremely important for authors to clearly understand and accurately represent the data being used, as confusion or mischaracterization can mislead readers and undermine the study’s credibility.
  - This inconsistency contributes to my recommendation to reject the manuscript.
- I have mentioned gravitational, rotational, and deformational (GRD) effects, however, the authors refer to GRD as Groundwater Recovery Depletion.
  - I have not encountered this term in the literature, so it is either a novel concept or I may be unfamiliar with it.
  - In either case, I kindly ask the authors to clarify what is meant by Groundwater Recovery Depletion, as it is highly relevant in this context. Providing a clear explanation and an appropriate reference would be very helpful.
- The description in Section 3.2 remains limited, despite this being raised in the first round of comments.
  - The effort to improve this section appears minimal. However, this section is critical for reader understanding, and the lack of detail disrupts the overall flow of the manuscript and does not meet publication standards.
  - I encourage the authors to provide a detailed description of the data sources used (e.g., TG stations, confidence levels), and explain why these sources were selected.
  - Although these elements may be mentioned throughout the article, but they should be consolidated within the relevant section to enhance clarity and coherence.
  - This is one of the reasons I am currently recommending rejection
- I previously requested (L151–152): “I do not see the value of this figure in the paper; consider replacing it.”
  - No justification has been provided, and both the figure and its caption remain limited.
  - The space could be better used for a sea level projection figure or an abbreviations table.

Many of the comments I am repeating here were mentioned previously (I have annotated the comments section in main PDF) and should have been fully addressed. I am therefore inclined to recommend rejection of the article at this stage. However, if the editor deems that the authors may resubmit, I would be happy to take a fresh look, provided:

- All comments are thoroughly addressed,

- The article is checked for typos, abbreviation use, and grammar
