# Supplementary material for: Assessment of the compound impact of sea level rise, land subsidence and storm surge under climate change in ShangHai
Source: PLoS One. 2026 Mar 18;21(3):e0343974. doi: 10.1371/journal.pone.0343974 (PMC12998868; doi:10.1371/journal.pone.0343974)
Supplement: Attachment — Submitted filename: Response to Reviewers.docx [file pone.0343974.s008.docx]

# R1 response to Reviewers' Comments

## Title

Assessment of the compound impact of sea level rise, land subsidence and storm surge under climate change.

**Authors**
Bing Liang, Guoqing Shi, Yuexi Wu, Yuxuan Zhu, Mark Wang, Zhonggen Sun

1. The grammar of the article requires further polishing (there are missing spaces).
   - I remain unconvinced that the use of abbreviations has been fully addressed. For instance, while sea level change is abbreviated as SLR, both SLR and sea level rise are used, which creates confusion.

Response： Thank you for your comments.Unfortunately, there are some places in the manuscript where not all abbreviations have been used. We have conducted a search of the entire text and ensured that all abbreviations have been used.

- Also, two figures were deleted, but I want to flag that Figure 4 is still mentioned in the text, which suggests the manuscript needs a more thorough review.

Response： Thank you for your comments.The original manuscript contained Figure 4, which was a graph plotted based on the data in Table 4. However, Figure 4 has been deleted, and we overlooked this revision. Now, Figure 4 has been revised to Table 4.

- This study is not yet adequate for use in planning, as it still does not provide uncertainty information needed for decision-making. Although the study is framed as a first attempt, the research limitations (Section 7) should be explicitly and clearly stated.
  - While the authors attempt to present uncertainty estimates, Tables 3, 4, and 5 do not incorporate uncertainty into the analysis. If confidence levels exist for the VLM data, I strongly recommend conducting a Monte Carlo simulation to meaningfully integrate sea-level data with VLM data. If this cannot be done, the authors must clearly state that the results should be interpreted with caution, particularly by those using the findings for planning purposes, due to the absence of quantified uncertainty.

Response： Thank you for your comments.We have added the following content in the "Research limitations" section:In our initial analysis, we added a deterministic subsidence rate to the median SLRprojection as a simplified first-order approximation to illustrate the potential compound effect.We acknowledge that this approach does not propagate the full uncertainty inherent in both theSLR projections and the subsidence estimate. Land subsidence prediction is intended to highlight the potential long-term cumulative effect if current subsidence mechanisms persist without mitigation. We fully agree that this approach has limitations. The projection does not account for potential future nonlinearities, changes in groundwater management policy, or other mitigating interventions that could alter the subsidence rate. These predicted values are uncertain values

- low-confidence sea level projections for SSP2-4.5.
  - I urge the authors to revisit the data sources carefully. In their response, the authors stated: *“By implementing these changes, our tables and results will more accurately reflect the deep uncertainty inherent in century-scale climate projections and local subsidence forecasts.”* However, deep uncertainty is typically associated with low-confidence sea level projections.
  - Since IPCC AR6 does not provide low-confidence sea level projections for SSP2-4.5, this suggests either a misunderstanding or that different datasets are being referenced.
  - The authors also claim to use high-confidence scenario (response to comment L158–159); however, I am not familiar with such sea level projection categories, and these do not appear to be available via the NASA Sea Level Change Portal.
  - It is extremely important for authors to clearly understand and accurately represent the data being used, as confusion or mischaracterization can mislead readers and undermine the study’s credibility.
- Response： Thank you for your comments.The data for sea level rise predictions are sourced from the IPCC AR6 dataset accessed via NASA’s Sea Level Change Portal , without any discussion on confidence levels.The data source is shown in the screenshot below：

- I have mentioned gravitational, rotational, and deformational (GRD) effects, however, the authors refer to GRD as Groundwater Recovery Depletion.
  - I have not encountered this term in the literature, so it is either a novel concept or I may be unfamiliar with it.
  - In either case, I kindly ask the authors to clarify what is meant by Groundwater Recovery Depletion, as it is highly relevant in this context. Providing a clear explanation and an appropriate reference would be very helpful.

Response： Thank you for your comments. Groundwater Recovery Depletion refers to the continuous depletion of groundwater due to insufficient recovery capacity after extraction. This expression is developed from groundwater depletion and is a new term. We have a misunderstanding of GRD you mentioned, and we have revised GRD to "Gravity, rotation, and deformation"effects.

- The description in Section 3.2 remains limited, despite this being raised in the first round of comments.
  - The effort to improve this section appears minimal. However, this section is critical for reader understanding, and the lack of detail disrupts the overall flow of the manuscript and does not meet publication standards.
  - I encourage the authors to provide a detailed description of the data sources used (e.g., TG stations, confidence levels), and explain why these sources were selected.
  - Although these elements may be mentioned throughout the article, but they should be consolidated within the relevant section to enhance clarity and coherence.

Response： Thank you for your comments. We have added the following content in the 3.2 section:

Since Working Group I of the IPCC AR6 has already made predictions on the rise in global sea level, this study adopts these predicted values for the following research. The IPCC AR6 predictions on SLR are based on the average sea level from 1995 to 2014 and are divided into two different scenarios: the Shared Socioeconomic Pathways (SSP) scenario and the Future Global Warming Levels scenario. The SSP is a new generation of scenario combinations constructed by the IPCC for comprehensive analysis of future climate change. These scenarios depict various different development patterns of future economic and social systems, reflecting the correlation between economic and social development and the challenges of mitigating and adapting to climate change. They are the core foundation for conducting climate change impact assessments and climate policy formulation. These include SSP1-1.9,SSP1-2.6, SSP2-4.5, SSP3-7.0, SSP5-8.5, and other scenarios. To highlight the research theme, which is the combined impact of SLR, land subsidence, and storm surges on coastal cities, and to reduce computational workload, we selected the sea-level rise prediction data under the medium carbon emission scenario of SSP2-4.5 for analysis. The prediction data was downloaded from the NASA Sea Level Change Portal.

The name of the tide gauge station we selected is "LUSI", which is located in China. The station is situated in Lvsigang Town, Rudong County, Jiangsu Province, and is one of the important tide gauge stations along the Yellow Sea coast, frequently used for research on sea level changes in China's coastal waters. The query website is: <https://sealevel.nasa.gov/ipcc-ar6-sea-level-projection-tool?psmsl_id=979&data_layer=scenario.>

- I previously requested (L151–152): “I do not see the value of this figure in the paper; consider replacing it.”
  - No justification has been provided, and both the figure and its caption remain limited.
  - The space could be better used for a sea level projection figure or an abbreviations table.

Response： Thank you for your comments. Figure 1 is a simple schematic diagram drawn based solely on the text content, representing the three scenarios, and carries no additional special meaning.We have already deleted it.

Special note: The comments in the PDF file have also been synchronously revised.
